# Design-Oriented Comparison of Si–Me (Me = Mo, Ti, Zr, Ta, W) Infiltration Coatings on C/C Sonotrodes for Ultrasonic Atomization of CuSn8: Microstructure, Phase Constitution, Wettability, Nanoindentation, and Process Performance

**DOI:** 10.3390/ma19132803

**Published:** 2026-07-01

**Authors:** Tomasz Choma, Mirosław Jakub Kruszewski, Aleksandra Chądzyńska, Bartosz Kalicki, Bartosz Morończyk, Jakub Ciftci, Łukasz Żrodowski, Joanna Zdunek, Marcin Leonowicz

**Affiliations:** 1 AMAZEMET Sp. z o. o. [Ltd.], 27 Jana Pawła II Ave., 00-867 Warsaw, Poland; tomasz.choma@amazemet.com (T.C.); aleksandra.chadzynska@amazemet.com (A.C.); bartosz.kalicki@amazemet.com (B.K.); bartosz.moronczyk@amazemet.com (B.M.); jakub.ciftci@amazemet.com (J.C.); lukasz.zrodowski@amazemet.com (Ł.Ż.); 2 Faculty of Materials Science and Engineering, Warsaw University of Technology, 141 Wołoska St., 02-507 Warsaw, Poland; joanna.zdunek@pw.edu.pl (J.Z.); marcin.leonowicz@pw.edu.pl (M.L.); 3 Department of Materials Science & Engineering, Carnegie Mellon University, Pittsburgh, PA 15213, USA

**Keywords:** composites, interfaces, carbon, silicides, functional applications

## Abstract

This study compares five Si–Me infiltration coatings, Si:Mo (1:4), Si:Ti (1:1), Si:Zr (1:5), Si:Ta (1:1), and Si:W (1:5), deposited on C/C sonotrodes for ultrasonic atomization of CuSn8. The coatings were evaluated in terms of phase constitution, microstructure, wettability, nanoindentation response, and powder-production performance. XRD showed that the coatings formed distinct multiphase reaction layers, with Si:Ta (1:1) being the most silicide-dominated system, while the other coatings contained carbide or silicide–carbide phases. Metallization strongly improved the surface wettability of C/C, especially for Si:Zr (1:5) and Si:W (1:5). Nanoindentation indicated the most favorable H/E* and H^3^/E*^2^ descriptors for Si:W (1:5) and Si:Mo (1:4). All coatings enabled high powder yields in single-run atomization tests, while apparent differences in particle-size distribution were observed among the coating conditions. Overall, the results show that coating selection for ultrasonic atomization should combine phase constitution, surface-state descriptors, near-surface mechanical response, layer retention, and process performance. Within the investigated conditions and the limitation of single-run atomization experiments, Si:W (1:5) emerged as the most promising and best-balanced coating candidate, while Si:Ta (1:1) and Si:Mo (1:4) remained relevant alternatives.

## 1. Introduction

Ultrasonic atomization produces highly flowable metallic powders by converting high-frequency vibration of a sonotrode into capillary-wave breakup at the liquid surface. Once the vibration amplitude exceeds a threshold, ligaments form at the crests of standing capillary waves and eject droplets whose size scales with frequency, amplitude, and liquid properties. Stable atomization therefore hinges on sustaining a thin, continuous liquid film on the vibrating surface under cyclic thermo-mechanical loads [1].

Carbon–carbon (C/C) composites are attractive sonotrode materials owing to their low density, high stiffness retention, and excellent thermal-shock resistance at elevated temperature. However, as-fabricated C/C is typically ill suited to prolonged contact with molten metals: (i) it is weakly wettable by many alloys, (ii) it is chemically vulnerable (e.g., oxidation in trace O2 and interfacial reactions), and (iii) local asperities concentrate stresses during vibration, promoting contact fatigue and surface damage. These factors undermine formation of a stable liquid film and degrade aerosolization repeatability. Improving interfacial behavior and near-surface robustness via surface engineering is therefore essential [2,3].

Silicon-containing infiltration coatings on C/C offer a promising route to address these limitations. Under high-temperature reactive processing, Si-based mixtures in contact with carbon may generate not only silicides, but also carbide and silicide–carbide phases, together with surface oxides formed during subsequent exposure to air. Prior work has demonstrated silicide-based and Ti–Si/MAX-related protection concepts for carbonaceous substrates; however, the literature still lacks a comparative, application-driven study linking the actual phase constitution of Si–Me coatings to wettability, near-surface mechanical response, and quantitative atomization metrics under a unified protocol [4,5,6,7].

Here, we address that gap by evaluating five infiltration-derived Si–Me coatings on C/C—Si:Mo, Si:Ti, Si:Zr, Si:Ta, and Si:W—and relating their materials attributes to process-level performance in ultrasonic atomization. In the present study, the coatings were characterized by SEM/EDS and XRD to establish not only continuity and morphology, but also the constitution of the resulting multiphase reaction layers formed on C/C. Contact-mechanical behavior is probed by instrumented indentation at 10 mN to obtain hardness (H) and reduced modulus (E*); from these we derive H/E* (a comparative descriptor related to elastic strain tolerance) and H^3^/E*^2^ (a comparative descriptor related to resistance to localized plastic deformation under concentrated contact loading), two widely used design descriptors for thin hard coatings [8,9,10].

Wettability is assessed by static water contact angles at room temperature; we report cos θ as a convenient, linearized measure of relative hydrophilicity across coatings prepared and measured identically. Because water has a higher surface tension than most molten Cu-based alloys and interfacial chemistry differs, cos θ (H_2_O) is used strictly as a comparative surface-state descriptor rather than an absolute predictor of metal wetting. This qualification is consistent with prior observations that wetting of Si-containing ceramics by metals depends strongly on surface condition, oxide films, and interfacial reactions [2,3].

Single-run atomization performance is quantified by sieve analysis of the produced powders, reporting the total yield and, crucially, the in-spec yield defined here as the mass fraction in the 45–150 µm range, an interval relevant to several powder-based processes (e.g., certain thermal-spray and directed-energy deposition feedstocks) and often used in practical specifications [11,12]. While optimal size windows depend on process and alloy, the focus on in-spec yield provides a practical, application-oriented metric for comparing single-run coating responses under identical atomization conditions.

Two considerations motivated the selection of Me = Mo, Ti, Zr, Ta, W. First, W and Mo form highly refractory disilicides (WSi_2_, MoSi_2_) with outstanding oxidation resistance at high temperature; such phases may contribute to favorable near-surface mechanical descriptors, though silica-rich scales and surface contamination can either help or hinder wetting depending on structure. Second, Ti, Zr, and Ta sample distinct silicide chemistries and oxygen affinities, offering diverse combinations of hardness, stiffness, and surface reactivity. The selected Si:Me ratios should be regarded as process-oriented coating formulations rather than as a compositionally normalized series designed to isolate only the effect of Me. By holding substrate, fabrication route (infiltration), and atomization protocol constant, we compare how selected coating formulations and resulting microstructures are associated with (a) room-temperature surface-state descriptors and (b) near-surface mechanical response—two prerequisites for sustaining a coherent liquid film that atomizes efficiently [4,5,6].

Our central hypothesis is that process-relevant performance—captured by in-spec yield and (where available) thickness retention—can be rationalized from a small set of coating-level descriptors, namely, H/E*, H^3^/E*^2^, and cos θ, provided the coating is morphologically continuous. We therefore interpret the coatings within an integrated comparative framework combining H/E*, H^3^/E*^2^, and cos θ, and examine how these descriptors relate to the measured single-run process outcomes. This compact representation distills practical design guidance for selecting Si–Me coatings that balance surface-state descriptors with near-surface mechanical response under ultrasonic loading [8,9,10].

## 2. Materials and Methods

### 2.1. Materials Fabrication and Atomization

The substrate was a C/C composite plate. Five different Si–Me coating systems were prepared on C/C by powder infiltration: Si:Mo (1:4, at.%), Si:Ta (1:1, at.%), Si:Ti (1:1, at.%), Si:W (1:5, at.%), and Si:Zr (1:5, at.%). The coating was deposited only on the working zone intended for ultrasonic atomization.

Infiltration was performed in a horizontal induction setup under flowing argon. The process was controlled by the applied induction current, heating time, argon atmosphere, and traverse sequence. The Si:Ti sequence comprised the following: (i) argon purge (~1 min), (ii) ramp at I ≈ 1200 A for ~150 s, (iii) traverse to the plate end, (iv) melt the layer and sweep from the plate end toward its center at I ≈ 1800 A for ~100 s, and (v) cool under argon (~3 min). For Si:W, Si:Mo, Si:Ta and Si:Zr the sequence was (i) argon purge (~1 min), (ii) traverse to the plate end, (iii) ramp at I ≈ 1820 A for ~150 s, (iv) uniform sweep at I ≈ 1800 A for ~100 s, and (v) cool under argon (~3 min). As the temperature was not directly recorded during infiltration, the process is reported in terms of controlled current–time profiles rather than visual heat-color descriptors.

Atomization was carried out on an induction module of rePowder system (AMAZEMET Sp. z o.o., Warsaw, Poland) equipped with a 40 kHz piezoelectric transducer, an ultrasonic amplitude booster, and a Nucleus DG1-1000 generator (NUCLEUS Ultraschall GmbH, Düsseldorf, Germany), with the C/C plate mounted horizontally between the booster and sonotrode.

For each coating condition, one atomization run was performed using a CuSn8 alloy melt (~500 g per run). A standard graphite nozzle Ø 0.5 mm was used. Atomization was conducted in an argon-purged chamber with the residual oxygen level stabilized at ~50 ppm. The melt was brought to ~1300 °C, requiring 6–6.5 min of heating to reach a fully fluid state. The ultrasonic transducer operated continuously at 40 kHz. Gas pressures were staged as follows: the melting stage was performed under a near-vacuum of 0.006 bar; prior to atomization the chamber was backfilled to 0.2 bar (Ar); atomization proceeded at a working gas pressure of 0.6 bar; and a short “turbo” boost of 1.5 bar was applied at initiation to ensure a clean, stable metal outflow before reverting to the 0.6 bar set-point. After cooling below ~400 °C, powders were collected from the cone and chamber walls for subsequent sieve analysis.

### 2.2. Characterization Methods

Phase identification of the as-infiltrated coatings was performed on a Bruker D8 Advance (DaVinci) diffractometer (Bruker AXS, Karlsruhe, Germany) using filtered Cu Kα radiation (λ = 1.5418 Å), 40 kV/35 mA, 2θ = 10–120°, step 0.05°, and 3 s per step in Bragg–Brentano geometry with a LynxEye strip detector (Bruker AXS, Karlsruhe, Germany). Patterns were analyzed in Diffrac.EVA (v6) against ICDD PDF-2.

Cross-section specimens were cut, mounted in a conductive resin, ground from P600 to P2500 grit and polished with 3 µm diamond suspension.

Microstructure and chemical composition were examined on a Thermo Scientific Axia ChemiSEM (Thermo Fisher Scientific Inc., Waltham, MA, USA) at 25 kV equipped with EDS. Both as-infiltrated and post-atomization states were analyzed.

Wettability was assessed by the sessile-drop method on a GoRame-Hart 90 Pro Edition goniometer (Ramé-hart Instrument co., Sandy, UT, USA) with manual micro-syringe dosing. Distilled water at room temperature was used; droplet volume 33.5 mm^3^ (~33.5 µL). For each drop, left and right contact angles were measured on at least 10 frames and averaged at the droplet level. The reported WCA for a given surface is the mean of three independent droplets, and the standard deviation refers to between-drop variability (n = 3). The corresponding cos θ values were calculated from the mean WCA and used later as a relative surface-state descriptor under identical surface histories.

Mechanical response of the coatings was measured on a Micromaterials Vantage Alpha with a Berkovich indenter (Micro Materials Ltd., Wrexham, UK). The load program used Pmax = 10 mN, 10 s loading/5 s hold/10 s unloading; thermal drift was assessed for 10 s after each cycle at <10% of Pmax. n = 3 indents per coating were performed. Curves were analyzed by the Oliver–Pharr method (with current tip-area calibration) to obtain hardness H and reduced modulus E*; derived indices (elastic work fraction, contact compliance/stiffness) were computed from the recorded datasets.

Post-atomization powders were sieved into discrete fractions, including the <20 µm bowl fraction and fractions later aggregated into the 45–150 µm target window, enabling determination of the particle size distribution (PSD), total powder yield (powder mass/feed mass), and the in-spec yield, defined here as the mass fraction in the 45–150 µm interval normalized to the feed mass. For each run, the feed mass, total collected powder mass, and the masses of the individual sieve fractions were recorded. Sieve fractions are reported throughout in interval form: 150+, 150–125, 125–100, 100–80, 80–63, 63–45, 45–20, and <20 µm. Because no dedicated 20–45 µm sieve fraction was available, the parameter termed Under63 proxy was calculated as the sum of the 63–45 µm fraction and the <20 µm bowl fraction; it is therefore treated as a lower-tail PSD surrogate rather than as the true cumulative fraction below 63 µm.

Coating thickness was estimated from the available cross-sectional SEM images by local line measurements taken perpendicular to the coating/substrate interface in regions where the coating boundaries were clearly identifiable. Because additional SEM examination could not be performed, the reported thickness values should be regarded as approximate local ranges from the examined fields rather than as statistically exhaustive thickness distributions.

## 3. Results and Discussion

### 3.1. Cross-Sectional Microstructure Before and After Atomization

**Figure 1:**
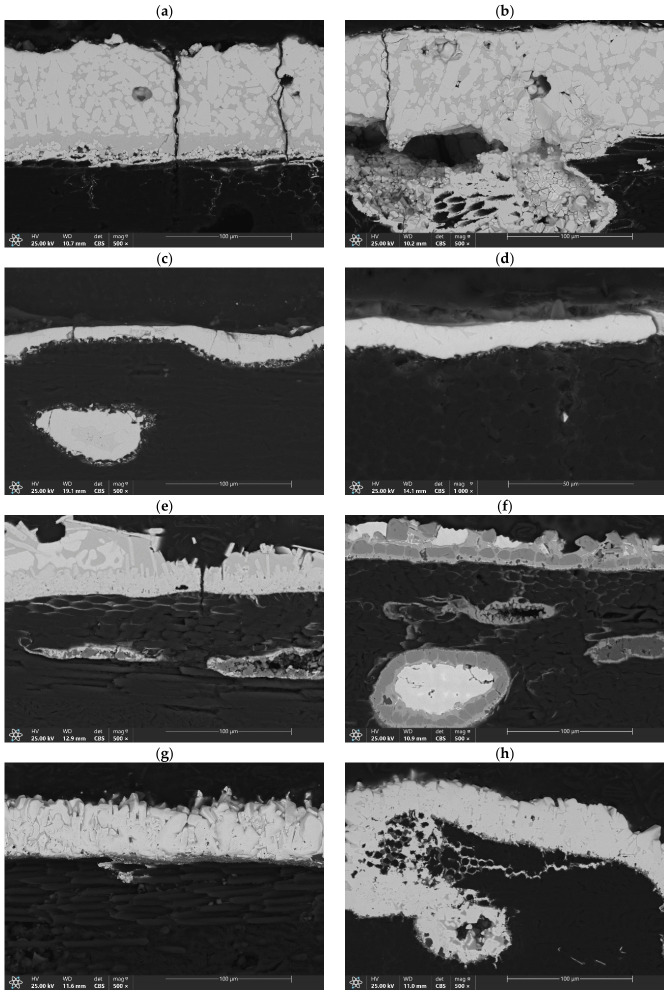

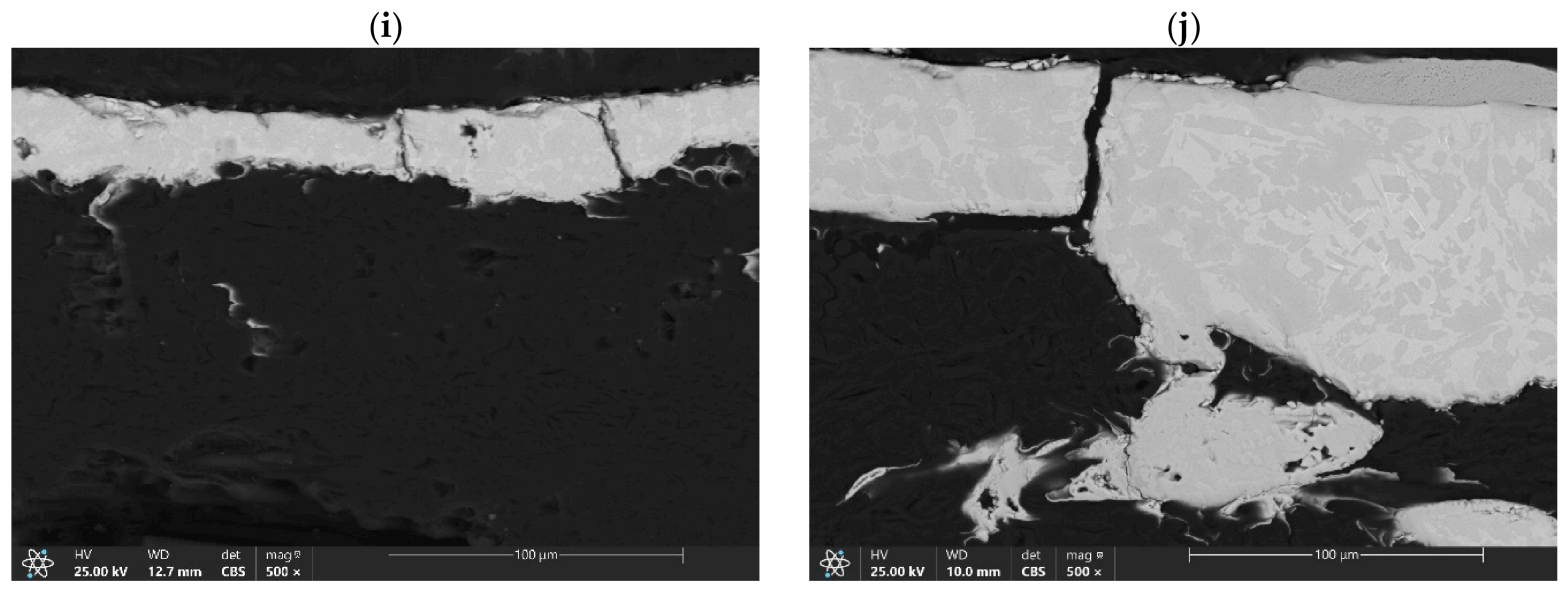
Cross-sectional SEM images of Si–Me infiltration coatings on C/C (Me = Ta, Mo, Ti, W, Zr) in the as-infiltrated state (left column) and after ultrasonic atomization (right column): (**a**,**b**) Si:Ta 1:1, (**c**,**d**) Si:Mo 1:4, (**e**,**f**) Si:Ti 1:1, (**g**,**h**) Si:W 1:5, (**i**,**j**) Si:Zr 1:5.

Figure 1 compares the cross-sectional SEM microstructure of the Si–Me infiltration coatings deposited on C/C in the as-fabricated condition and after a full ultrasonic atomization run. In all investigated systems, the coating remained clearly distinguishable from the underlying carbon–carbon composite architecture, which enabled a first-order assessment of coating continuity, local thickness uniformity, cracking, and the extent of process-induced morphological alteration in the examined cross-sections under coupled thermal, vibrational, and melt-contact loading. In the present section, the SEM observations are interpreted conservatively in morphological terms, whereas phase-level assignments are based on XRD analysis and are further correlated with SEM/EDS elemental-distribution maps [1,4,5,6,7].

The Si:Ta (1:1) coating (Figure 1a,b) formed a macroscopically continuous layer in the as-fabricated condition, with a comparatively uniform appearance across the imaged cross-section. The thickness was approximately 100 µm, and several cracks propagating roughly perpendicular to the surface were observed. After atomization, the coating remained present and continuous in the examined region, without evidence of large-area spallation or catastrophic delamination. Despite repeated thermal cycling and interaction with the molten CuSn8 alloy, no major morphological changes were evident, which points to high structural stability of the layer during ultrasonic operation. This retention is also consistent with the low dispersion observed in the wettability data, suggesting a comparatively homogeneous outer-surface state. The SEM/EDS elemental-distribution maps for the as-infiltrated and post-atomization states are presented in Figures S1 and S2, respectively. In both states, the maps show a continuous Si–Ta-rich reaction layer and do not reveal pronounced elemental redistribution or local loss of coating continuity after atomization. These observations support the interpretation that the Si:Ta coating retained a comparatively homogeneous working layer in the examined region under the applied process conditions.

The Si:Mo (1:4) system (Figure 1c,d) was distinctly thinner, with a typical thickness of approximately 10–30 µm, and exhibited a relatively uniform cross-sectional contrast in the as-fabricated state. After atomization, the coating remained detectable and no major disruption of the layer was observed in the examined area. This observation is important because Si:Mo behaves as a wettability outlier among the metallized systems; although its water contact angle is substantially higher than those of Si:Zr, Si:W, Si:Ti, and Si:Ta, the preserved coating integrity suggests that this system may still remain functionally relevant if near-surface mechanical response and coating retention under the applied atomization conditions are prioritized over initial room-temperature surface affinity alone. The SEM/EDS elemental-distribution maps for the as-infiltrated and post-atomization states are presented in Figures S3 and S4, respectively. In both states, the maps show a continuous Mo–Si-containing reaction layer and do not reveal pronounced changes in the elemental distribution after atomization. No evident coating damage, local discontinuity, or defect formation is observed in the mapped region. These observations support the interpretation that, despite its relatively limited thickness, the Si:Mo coating retained a continuous working layer in the examined region under the applied process conditions.

The Si:Ti (1:1) coating (Figure 1e,f), with a typical thickness of approximately 30–50 µm in the as-fabricated condition, also formed a continuous layer, but appeared more spatially heterogeneous than the Ta- and Mo-containing systems. After atomization, the coating was still present; however, the near-surface region showed clearer signs of modification, including a reduced apparent thickness (approximately 10–40 µm) and increased heterogeneity. At this stage, these changes are described conservatively as local restructuring and morphological alteration rather than as confirmed phase transformation, because phase-level identification requires support from XRD and SEM/EDS data. Nevertheless, the Ti-containing coating appears to be more susceptible to process-induced modification than the Ta-, Mo-, or W-based systems. The SEM/EDS elemental-distribution maps for the as-infiltrated and post-atomization states are presented in Figures S5 and S6, respectively. In the as-infiltrated state, the maps reveal local variations in the Si and Ti distributions, which are consistent with the heterogeneous character of the Ti-containing reaction layer and may reflect the formation of Ti–Si- and/or Ti–Si–C-based constituents identified by XRD. After atomization, the mapped region shows a modified near-surface morphology, with a weaker Si signal while the Ti-rich regions remain clearly visible. In addition, the Cu signal is detected not only at the coating surface but also within the underlying sonotrode material, which is consistent with possible local penetration or retention of Cu-bearing material within the C/C substrate. These observations are also in line with the interpretation that the Si:Ti coating was more susceptible to local process-induced modification under the applied atomization conditions.

The Si:W (1:5) coating (Figure 1g,h) formed a continuous layer with a comparatively coherent morphology already in the as-fabricated state, with a thickness of approximately 50 µm. After atomization, the coating remained clearly visible across the section, indicating good thickness retention and resistance to catastrophic delamination. In combination with the highest nanoindentation-derived mechanical indices reported for this system, the preserved cross-sectional morphology supports the interpretation that the W-containing coating is among the most promising candidates in the present comparative framework, where contact- and vibration-assisted degradation are expected to be critical failure drivers [4,6,8,9,10]. The SEM/EDS elemental-distribution maps for the as-infiltrated and post-atomization states are presented in Figures S7 and S8, respectively. In both states, the maps show a continuous W–Si-containing reaction layer with a qualitatively similar elemental distribution. No pronounced elemental redistribution, local discontinuity, or coating degradation is visible in the mapped region after atomization. These observations support the interpretation that the Si:W coating retained a continuous working layer under the applied process conditions.

The Si:Zr (1:5) system (Figure 1i,j) showed a continuous coating character with visible local cracks and a relatively broad thickness range of approximately 10–100 µm. Despite this variability, the layer remained present after atomization and the coating/substrate interface was still clearly identifiable, without evident large-scale disruption in the analyzed region. This behavior suggests satisfactory structural continuity under the imposed process conditions. From a design viewpoint, it is also notable that Si:Zr showed the lowest water contact angle among all investigated surfaces, making it an especially interesting case in which highly favorable surface affinity coexists with acceptable post-process coating retention. The SEM/EDS elemental-distribution maps for the as-infiltrated and post-atomization states are presented in Figures S9 and S10, respectively. In both states, the maps show a broadly similar distribution of Si and Zr across the reaction layer, without a pronounced change in the spatial arrangement of these elements after atomization. These observations indicate that the applied atomization conditions did not lead to an evident change in the Si–Zr elemental-distribution pattern in the mapped region.

Taken together, the cross-sectional observations show that the investigated coatings do not respond equally to the combined thermal and vibrational loading of ultrasonic atomization. Coatings such as Si:W, Si:Ta, Si:Mo, and, to a slightly more qualified extent, Si:Zr retained a continuous working layer in the examined cross-sections after processing, whereas Si:Ti exhibited more pronounced local modification of the near-surface region. In practical terms, such coatings are expected to provide more favorable conditions for maintaining a stable molten film, although atomization reproducibility cannot be inferred from morphology alone [1]. At the same time, morphological continuity alone is not sufficient for coating selection and must be interpreted together with phase constitution, surface-state descriptors, and contact-mechanical response.

### 3.2. Phase Constitution of the As-Fabricated Coatings

The phase constitution of the as-fabricated Si–Me coatings was examined by X-ray diffraction, while the full diffraction patterns and phase indexing are provided in the Supplementary Materials (Figures S12–S16). Because the coatings were formed by reactive processing of Si–Me powder mixtures in direct contact with a C/C substrate, the resulting phase assemblages are expected to reflect not only the nominal Si:Me ratio, but also interaction with carbon and, in some cases, partial surface oxidation.

It should be noted that the XRD-based phase assignments have inherent limitations in the present multiphase coatings. Peak overlap between silicide-, carbide-, oxide-, and carbon-related reflections may complicate unambiguous identification of minor phases, particularly when phases are present in low amounts or exhibit different crystallinity. Moreover, the Bragg–Brentano geometry provides information averaged over the diffracting volume and does not resolve the through-thickness or near-surface distribution of the detected phases. Therefore, the XRD results are interpreted together with the SEM/EDS elemental-distribution maps presented in Figures S1–S10, which provide complementary information on the spatial distribution of the main elements but are not used as standalone crystallographic phase-identification evidence.

The XRD results indicate that all investigated coatings developed phase-distinct reaction layers, although their phase constitution differed markedly from system to system. The Si:Zr (1:5) coating (Figure S12) was indexed to Zr_2_Si, ZrC, ZrO_2_, and SiO_2_, indicating a multiphase reaction layer formed through concurrent silicide formation, carbide formation, and oxidation-related processes. The coexistence of zirconium silicide and zirconium carbide is consistent with carbon participation from the C/C substrate during coating formation, while the oxide phases point to the presence of oxidized constituents within the analyzed volume.

The Si:Ta (1:1) coating (Figure S13) was dominated by Ta_5_Si_3_, making it the most clearly silicide-dominated system among the investigated coatings. In contrast to the other systems, no carbide phase was resolved in the XRD pattern, which suggests that the Ta-containing layer was dominated by tantalum silicide formation within the diffracting volume.

For the Si:Mo (1:4) coating (Figure S14), the detected phases were C, SiC, MoC, and Mo_4.8_Si_3_C_0.6_. This phase assemblage indicates that the Mo-containing coating did not develop as a simple binary silicide layer but, rather, as a mixed carbide/silicide–carbide system in which carbon participation played an important role. The simultaneous presence of SiC, Mo carbide, and a ternary Mo–Si–C phase points to coupled reaction pathways involving both silicon and carbon during coating formation.

The Si:Ti (1:1) coating (Figure S15) was indexed to Ti_3_Si, Ti_3_SiC_2_, and SiO_2_. The presence of the ternary Ti_3_SiC_2_ phase is consistent with carbon involvement in the phase evolution of the coating. The Ti-containing system should therefore be regarded as a mixed silicide/silicon–titanium carbide-derived layer rather than as a purely Ti–Si silicide coating. The detected silica phase suggests an additional oxidized surface contribution. The heterogeneous Ti- and Si-distribution observed in the SEM/EDS maps of the as-infiltrated coating is consistent with the multiphase Ti–Si/Ti–Si–C character inferred from XRD.

The Si:W (1:5) coating (Figure S16) contained WC, W_5_Si_3_, SiO_2_, and C. As in the Mo- and Ti-containing systems, the XRD results indicate simultaneous operation of silicide-forming and carbide-forming reaction pathways. The coexistence of W_5_Si_3_ and WC suggests that both Si–W and C–W interactions contributed to the final coating architecture, whereas the presence of SiO_2_ indicates an oxidized component within the analyzed volume.

Taken together, the XRD results show that the investigated Si–Me coatings should not be treated as a uniform family of simple binary silicides. Instead, they constitute system-specific multiphase reaction layers, whose constitution depends strongly on the choice of metal and on the extent of interaction with the carbon substrate. A particularly important outcome is that carbon participation is indicated in the Mo, Ti, W, and Zr systems, where carbide or silicide–carbide phases were identified, whereas the Ta-containing coating appears to be the most distinctly silicide-based.

These phase differences are relevant for interpreting the functional behavior discussed in the following sections. First, the nanoindentation response reflects the effective mechanical behavior of real multiphase coatings rather than phase-pure bulk compounds. Second, the room-temperature water contact-angle response may be influenced by the near-surface chemical state, including the presence of silicide-, carbide-, and oxide-containing constituents; however, XRD alone does not determine the actual outermost surface chemistry. At the same time, the XRD results should be interpreted together with SEM/EDS observations, because diffraction alone does not resolve how the detected phases are distributed through the coating thickness or localized near the outer surface.

Overall, the XRD analysis confirms that the as-fabricated coatings formed chemically distinct working surfaces on C/C, and that these differences are sufficiently pronounced to justify subsequent comparison in terms of surface-state descriptors, near-surface mechanical response, and atomization-related process performance.

### 3.3. Wettability

Wettability of the pristine C/C substrate and the metallized Si–Me-coated surfaces was assessed via static water contact angle (WCA) measurements at room temperature (sessile-drop method; see Section 2.2). Water WCA is used here only as a comparative descriptor of the room-temperature surface state under identical surface histories and test conditions. It should not be interpreted as a direct predictor of molten CuSn8 wetting, because high-temperature metal wetting is governed by alloy surface tension, oxide films, interfacial reactions, and kinetic effects that are not reproduced in the water-based test [2,3]. The results are summarized in Table 1.

The uncoated C/C exhibits a hydrophobic response, with WCA = 101.0 ± 6.5°. Metallization leads to a pronounced decrease in WCA for four of the five coatings, shifting the water-based surface response toward a strongly hydrophilic regime: Si:Zr 1:5 (15.8 ± 4.7°), Si:W 1:5 (20.4 ± 7.0°), Si:Ti 1:1 (27.1 ± 10.8°), and Si:Ta 1:1 (27.4 ± 3.1°). Expressed as cos θ (used here only for a linearized comparison of relative hydrophilicity), these correspond to approximately −0.19 (C/C) versus 0.89–0.96 for the most hydrophilic metallized systems (Si:Ti/Si:Ta ≈ 0.89; Si:W ≈ 0.94; Si:Zr ≈ 0.96). In practical terms, this indicates that Si–Me metallization substantially alters the near-surface state relative to pristine C/C, most likely through changes in oxide chemistry, surface polarity, and local topography developed after coating formation and air exposure [4,5,6].

A clear compositional outlier is Si:Mo 1:4, which remains only moderately wettable (70.7 ± 4.8°; cos θ ≈ 0.33). This still represents a substantial improvement relative to bare C/C, but it suggests that the Mo-containing surface presents an outermost state that is less hydrophilic than the other Si–Me coatings. This difference can be discussed conservatively in two non-exclusive ways: (a) surface chemistry— the outermost region may contain constituents or oxide/contamination states with lower polarity; and/or (b) surface heterogeneity/roughness—a heterogeneous surface can increase the apparent WCA via mixed wetting states, while simultaneously increasing measurement scatter [2,3]. Importantly, the magnitude of the standard deviations provides a first hint of uniformity: Si:Ta 1:1 shows low dispersion (±3.1°), consistent with a highly uniform surface state at the droplet footprint scale, whereas Si:Ti 1:1 shows the largest scatter (±10.8°), which is compatible with stronger local variability (e.g., partial coverage, mixed surface phases, microcracking/porosity, or pronounced roughness gradients). These interpretations are consistent with the cross-sectional evidence already presented in Section 3.1. In particular, the low WCA dispersion of Si:Ta is in line with its comparatively uniform and morphologically stable coating, whereas the much larger scatter observed for Si:Ti is consistent with the stronger heterogeneity and post-atomization modification visible in cross-section. For Si:Mo, the combination of a comparatively high WCA with preserved coating continuity indicates that a less hydrophilic outer-surface state does not necessarily preclude functional relevance when coating retention and near-surface mechanical response are also considered. Accordingly, the wettability trends are best interpreted as comparative indicators of surface condition rather than as direct predictors of atomization performance.

From a process-design standpoint, the surface state of the sonotrode is relevant because ultrasonic atomization relies on the formation and maintenance of a molten-metal film on the vibrating surface [1]. In general, insufficient melt–surface affinity may contribute to unstable liquid-film coverage; however, the present water-based WCA measurements cannot directly establish such behavior for molten CuSn8. In this context, the shift toward lower water contact angles for Si:Zr, Si:W, Si:Ti, and Si:Ta confirms that metallization modifies the room-temperature surface state, consistent with its intended role as an interface-conditioning step. Nevertheless, any correlation between water WCA and atomization outcome (yield, in-spec fraction) should be interpreted cautiously, because wetting and spreading of liquid metals are governed by high-temperature interfacial reactions, oxide formation, alloy surface tension, and kinetic constraints that are not captured by room-temperature water measurements [2,3]. Therefore, WCA is treated here only as a comparative surface-state descriptor and not as mechanistic evidence of molten CuSn8 wetting or atomization stability.

Finally, it is useful to view wettability alongside the other property sets reported here (nanoindentation indices and sieve-based PSD/yield). WCA primarily reflects the room-temperature surface state, whereas H/E* and H^3^/E*^2^ are used as comparative near-surface mechanical descriptors related to elastic strain tolerance and resistance to localized plastic deformation under the same testing conditions (see Section 3.4). These attributes are complementary: a promising coating candidate within the present comparative framework should combine a surface state conducive to sustaining a molten film (low WCA, high cos θ) with favorable near-surface mechanical descriptors, such as H/E* and H^3^/E*^2^. The present results already suggest that such a multicriteria assessment is necessary, as exemplified by the Mo-containing system: despite comparatively higher WCA, it can still be competitive if its mechanical indicators and post-atomization integrity remain favorable. The SEM/EDS observations discussed in Section 3.1 provide additional context for these WCA trends by linking the measured surface response with coating morphology and elemental-distribution patterns.

### 3.4. Mechanical Properties

**Figure 2:**
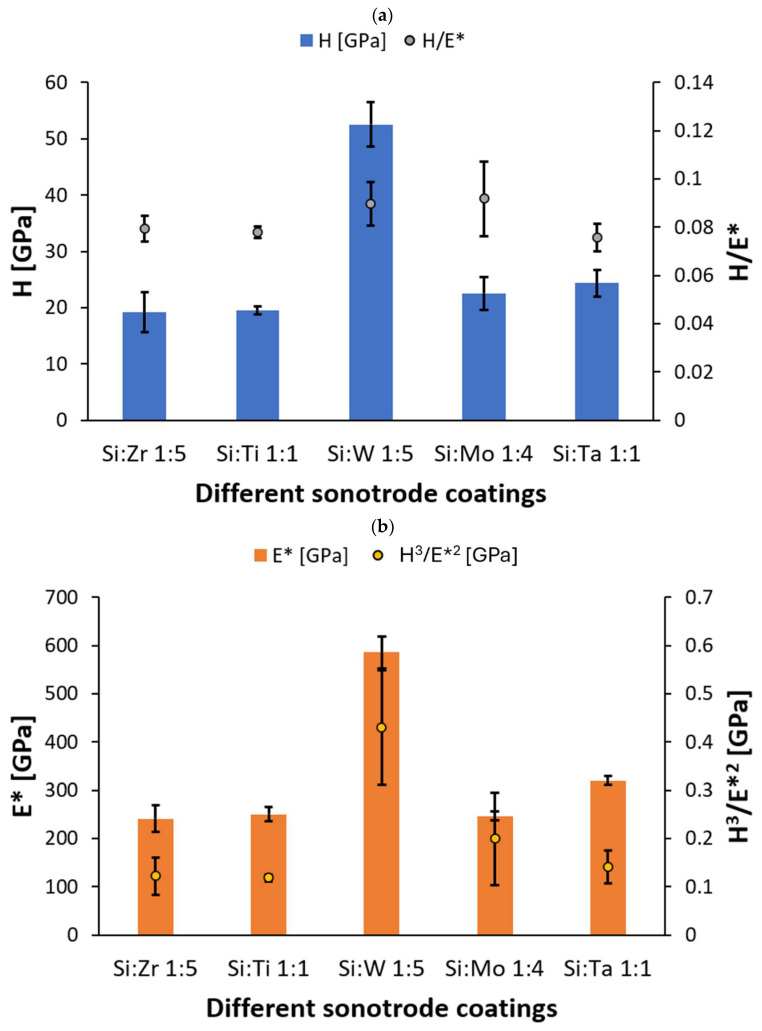
Nanoindentation-derived mechanical descriptors of the investigated Si–Me coatings on C/C: (**a**) hardness (H) and H/E*, and (**b**) reduced modulus (E*) and H^3^/E*^2^. Data are presented as mean ± standard deviation based on three indents per coating.

Figure 2 summarizes the nanoindentation-derived mechanical descriptors of the investigated Si–Me coatings. For improved readability, the data are shown in two panels: Figure 2a presents hardness (H) together with H/E*, whereas Figure 2b presents reduced modulus (E*) together with H^3^/E*^2^. Error bars indicate standard deviations calculated from three indents per coating.

Nanoindentation revealed marked differences in the mechanical response of the coatings. The Zr- and Ti-containing layers exhibited comparable hardness of approximately 18–20 GPa and reduced modulus E* of roughly 230–250 GPa, whereas the Ta- and especially W-containing coatings were distinctly stiffer and harder, with Si:Ta (1:1) reaching approximately 25 GPa/320 GPa and Si:W (1:5) approaching 50 GPa at E* close to 580–600 GPa. This trend is qualitatively consistent with the effective stiffness of the phase assemblages identified by XRD, particularly the W_5_Si_3_/WC-containing and Ta_5_Si_3_-dominated coatings, although the present nanoindentation response reflects real multiphase layers rather than phase-pure bulk compounds.

To compare the coatings on a more functional basis, the data were recast into the derived descriptors H/E* and H^3^/E*^2^. The H/E* ratio is dimensionless and is commonly used as an indicator related to elastic strain tolerance, whereas H^3^/E*^2^ has units of stress and is used here as a comparative descriptor related to resistance to localized plastic deformation under concentrated contact loading [8]. All coatings fall in the regime H/E* ≈ 0.08–0.13, i.e., around or above the often-quoted threshold of H/E* ≈ 0.1 associated with improved damage tolerance and wear resistance in hard and nanocomposite coatings [10,13]. However, these values should be interpreted only as comparative descriptors within the present experimental framework, not as direct measures of fracture toughness, contact-damage resistance, or long-term durability. The Si:W (1:5) and Si:Mo (1:4) systems exhibit the highest H/E* and H^3^/E*^2^ values among the investigated coatings, indicating a more favorable near-surface mechanical response under the applied indentation conditions. In contrast, the Si:Zr (1:5) and Si:Ti (1:1) coatings show lower H^3^/E*^2^ despite similar H/E*, suggesting comparatively lower resistance to localized plastic deformation under concentrated contact loading. These differences are relevant for the design-oriented comparison of the coatings, but they should not be interpreted as a direct prediction of degradation behavior during ultrasonic atomization.

It should be noted that H/E* and H^3^/E*^2^ cannot be interpreted as direct proxies for fracture toughness in all coating systems, as emphasized in recent critical assessments [9]. Nevertheless, when used comparatively within a single experimental framework, they provide a useful ranking of the investigated coatings in terms of their near-surface mechanical response. In the present case, the nanoindentation results indicate that the W- and Mo-containing reaction layers show the most favorable combination of H/E* and H^3^/E*^2^, whereas the Zr- and Ti-containing coatings appear less favorable in terms of these nanoindentation-derived descriptors. These results are therefore treated as one element of the multicriteria coating assessment, together with phase constitution, coating morphology, elemental distribution, wettability descriptors, and atomization-related process outcomes.

### 3.5. Powder Size Distribution and Process Performance

**Figure 3:**
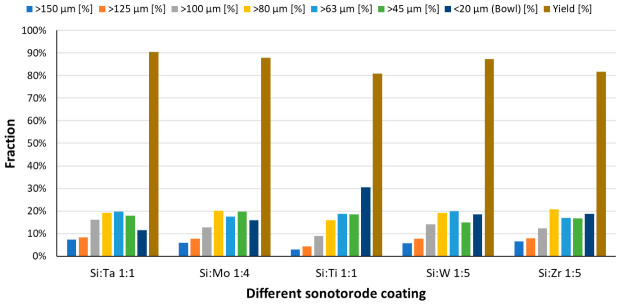
Sieve-based particle size distribution (PSD) and total powder yield for CuSn8 powders produced by ultrasonic atomization using Si–Me-coated C/C sonotrodes. Fractions are reported as 150+, 150–125, 125–100, 100–80, 80–63, 63–45, and <20 µm, together with total yield. PSD and yield values correspond to one atomization run per coating condition; therefore, no run-to-run error bars are shown.

Figure 3 summarizes the sieve-based PSD and total powder yield obtained after one ultrasonic atomization run per coating condition using the investigated Si–Me-coated C/C sonotrodes. To distinguish overall powder generation from application-oriented size selection, three process descriptors are used in the present work: (a) total yield, defined as powder mass divided by feed mass; (b) in-spec yield, defined here as the mass fraction in the 45–150 µm interval normalized to the feed mass; and (c) the Under63 proxy, used only as a lower-tail PSD indicator. This distinction is important because the primary process indicator in the present study is not the cumulative fine-powder fraction, but the ability of a given coating to deliver a favorable targeted fraction under otherwise identical atomization conditions. Because each coating condition was evaluated in a single atomization run, the PSD and yield values are interpreted as single-run process indicators rather than as statistically validated measures of coating-dependent atomization performance.

All systems produced broad multimodal PSDs spanning from <20 µm to >150 µm, with atomization yields in the range of approximately 80–90 wt.% of the feed mass. These yields are comparable to values reported for cold-crucible and ultrasound-assisted atomization routes developed for metallic powder production [14,15]. For all compositions, the fraction of oversized particles > 150 µm remained limited, while the combined 63–150 µm range accounted for a substantial share of the collected mass. The content of very fine powder < 20 µm was moderate, indicating that none of the investigated coatings shifted the single-run process outcome toward an excessively fine-dominated regime.

Despite the single-run character of the atomization tests, apparent differences in PSD shape were observed among the coating conditions. The Si:Ti (1:1) coating generated a broader and less centered distribution, with a noticeably higher share of fines and of the >100 µm fraction. By contrast, Si:Mo (1:4) and Si:W (1:5) showed a more even distribution between 63 and 125 µm, whereas Si:Ta (1:1) and Si:Zr (1:5) yielded somewhat higher contributions in the intermediate 80–100 µm classes. These differences suggest that coating condition may influence the molten-film breakup regime and the resulting particle-size statistics; however, replicate atomization experiments would be required to confirm the statistical significance of these trends [15,16,17].

Because no dedicated 20–45 µm sieve fraction was available, the lower tail of the PSD is discussed here using the Under63 proxy, calculated as the sum of the 63–45 µm fraction and the <20 µm bowl fraction. This parameter is therefore not interpreted as the true cumulative fraction below 63 µm, but only as a practical surrogate describing the tendency of a given coating to shift the PSD toward the fine-particle side. The primary size-selection metric remains the in-spec yield in the 45–150 µm interval.

From a practical standpoint, the most favorable coating is not necessarily the one that maximizes a single fraction in isolation but, rather, the one that offers the best balance between targeted-size production, limited excessive fines, and robust overall powder recovery. This distinction is important because a coating may show favorable surface-state descriptors but still produce a less favorable PSD/yield response if other factors, such as coating retention or near-surface mechanical response, are less advantageous. Conversely, a coating with less favorable room-temperature WCA may remain relevant if it retains continuity and exhibits favorable near-surface mechanical descriptors.

If the resulting powders are discussed in the broader context of downstream processability, the obtained PSDs fall within, or close to, ranges commonly considered usable in powder-based manufacturing routes, where particle-size distribution affects flowability, packing behavior, and process stability [16,17,18,19]. In the present study, however, such considerations remain secondary to the main objective, namely screening which coating systems provide the most promising single-run atomization outcomes under a fixed ultrasonic processing window.

### 3.6. Integrated Framework for Coating Selection

Ultrasonic atomization relies on the formation of capillary/Faraday waves on a thin liquid layer and subsequent ejection of droplets from the vibrating surface; hence, the stability of the melt film on the sonotrode and the integrity of the working coating under vibration-, thermal-, and contact-related loading are expected to influence powder size distribution and process yield. Recent in situ observations of ultrasonic atomization highlight that atomization dynamics is governed by the evolution of capillary waves and their interaction with cavitation-driven disturbances, which ultimately dictate droplet detachment and particle-size statistics [1]. Early foundational work also established ultrasonic atomization as a viable route for producing metallic powders by vibrating a liquid layer on a horn- or plate-like emitter [20].

In this context, the measured room-temperature water contact angle is used only as a comparative descriptor of the surface state, rather than as a direct measure of molten CuSn8 wetting. Lower WCA and higher cos θ indicate higher relative hydrophilicity in the water-based test, but they do not directly establish the ability of molten CuSn8 to form or maintain a continuous film during atomization. Complementary to this surface-state descriptor, nanoindentation-derived metrics provide a compact comparative description of how the metallized surface may tolerate localized mechanical interactions under high-frequency excitation. In particular, H/E* is widely used as an indicator related to elastic strain tolerance in contact, while H^3^/E*^2^ is interpreted here as a comparative measure related to resistance to plastic deformation under concentrated loading [8,10]. These descriptors are not treated as direct measures of fracture toughness, contact-damage resistance, or long-term durability. Moreover, the partitioning of indentation work into elastic and plastic components is not independent of H/E*, and can be rationalized using established energetic relationships for instrumented indentation [21].

Accordingly, the coatings can be compared within an integrated framework combining three levels of evidence: (i) surface state, represented by WCA/cos θ; (ii) near-surface mechanical response, represented by H/E* and H^3^/E*^2^; and (iii) single-run process outcome, represented by in-spec yield, total yield, and the Under63 proxy. Within this framework, the most promising coatings are expected to combine favorable surface-state descriptors with favorable nanoindentation-derived mechanical descriptors, retained coating continuity, and promising process response under the applied atomization conditions. By contrast, coatings with favorable water-WCA values but less favorable mechanical descriptors or poorer coating retention may not necessarily provide the most advantageous process outcome. Conversely, coatings with less favorable room-temperature WCA may remain relevant if they retain continuity and exhibit favorable near-surface mechanical descriptors.

Applied to the present dataset, including the single-run atomization outcomes, this integrated interpretation suggests that Si:W (1:5) is the most promising and best-balanced coating candidate within the investigated conditions. It combines low water WCA, favorable nanoindentation-derived mechanical descriptors, and good morphological retention after atomization. Si:Ta (1:1) appears to be a robust and comparatively homogeneous alternative within the present comparison, especially in view of its low WCA dispersion and stable cross-sectional morphology. Si:Zr (1:5) stands out primarily through its very low water WCA and remains a relevant candidate when surface-state descriptors are prioritized. Si:Mo (1:4), in turn, behaves as a functional outlier: despite its substantially higher WCA, it remains potentially competitive because of its favorable nanoindentation-derived mechanical descriptors and preserved coating continuity after atomization. By contrast, Si:Ti (1:1) appears less favorable within the present multicriteria comparison, because its relatively favorable wettability is offset by greater heterogeneity and more pronounced post-process modification of the working layer.

The main value of this framework is that it links surface state, near-surface mechanical response, coating continuity, and process outcome into a single decision-oriented interpretation. In this sense, the present results indicate that coating selection for ultrasonic atomization should not be based on wettability, hardness, or yield alone, but on the combined assessment of phase constitution, elemental distribution, coating morphology, surface-state descriptors, nanoindentation-derived mechanical response, and single-run PSD/yield response. Because the atomization experiments were performed as one run per coating condition, the process-related part of this framework should be treated as comparative screening rather than as a statistically validated ranking of coating performance.

## 4. Conclusions

Five Si–Me infiltration coatings on C/C were compared as candidate working surfaces for ultrasonic atomization of CuSn8. The investigated systems formed distinct multiphase reaction layers rather than a uniform family of binary silicides. XRD indicated a predominantly silicide-based character for Si:Ta (1:1), whereas the Mo-, Ti-, W-, and Zr-containing coatings exhibited phase assemblages consistent with carbide and/or silicide–carbide participation, together with oxide-containing constituents in the analyzed volume.

The results show that coating selection should rely on a combined assessment of phase constitution, elemental distribution, coating morphology, surface-state descriptors, nanoindentation-derived mechanical response, and process outcome. Within the investigated processing conditions and the limitation of single-run atomization experiments, Si:W (1:5) emerged as the most promising and best-balanced coating candidate, combining low water WCA, favorable H/E* and H^3^/E*^2^ descriptors, and good post-atomization morphological retention in the examined cross-sections. Si:Ta (1:1) represents a robust and comparatively homogeneous alternative, whereas Si:Mo (1:4) remains a relevant outlier whose lower room-temperature hydrophilicity is partly offset by favorable mechanical descriptors and preserved coating continuity. Further replicated atomization experiments would be required to confirm the statistical significance of the observed PSD/yield trends.

## Figures and Tables

**Table 1:** Static water contact angle and derived surface-state metrics for pristine C/C and Si–Me metallized C/C surfaces. WCA values are reported as mean ± standard deviation based on three independent droplets.

Material	WCA, θ [°]	± [°]	± [%]	cos θ
C/C	101	6.5	6.4	−0.191
Si:Ti (1:1)	27.1	10.8	39.9	0.891
Si:W (1:5)	20.4	7	34.3	0.937
Si:Zr (1:5)	15.8	4.7	29.8	0.962
Si:Ta (1:1)	27.4	3.1	11.3	0.887
Si:Mo (1:4)	70.7	4.8	6.8	0.33

## Data Availability

The original contributions presented in this study are included in the article/Supplementary Materials. Further inquiries can be directed to the corresponding author.

## Supplementary Materials

The following supporting information can be downloaded at: https://www.mdpi.com/article/10.3390/ma19132803/s1, Figure S1: Elemental mapping Si:Ta 1:1 after infiltration; Figure S2: Elemental mapping Si:Ta 1:1 after atomization; Figure S3: Elemental mapping Si:Mo 1:4 after infiltration; Figure S4: Elemental mapping Si:Mo 1:4 after atomization; Figure S5: Elemental mapping Si:Ti 1:1 after atomization; Figure S6: Elemental mapping Si:Ti 1:1 after infiltration; Figure S7: Elemental mapping Si:W 1:5 after infiltration; Figure S8: Elemental mapping Si:W 1:5 after atomization; Figure S9: Elemental mapping Si:Zr 1:5 after infiltration; Figure S10: Elemental mapping Si:Zr 1:5 after atomization; Figure S11: XRD patterns of C/C substrate before infiltration; Figure S12: XRD patterns of Si:Zr 1:5 after infiltration; Figure S13: XRD patterns of Si:Ta 1:5 after infiltration; Figure S14: XRD patterns of Si:Mo 1:4 after infiltration; Figure S15: XRD patterns of Si:Ti 1:1 after infiltration; Figure S16: XRD patterns of Si:W 1:5 after infiltration.
