# Supplementary material for: Design-Oriented Comparison of Si–Me (Me = Mo, Ti, Zr, Ta, W) Infiltration Coatings on C/C Sonotrodes for Ultrasonic Atomization of CuSn8: Microstructure, Phase Constitution, Wettability, Nanoindentation, and Process Performance
Source: Materials (Basel). 2026 Jul 1;19(13):2803. doi: 10.3390/ma19132803 (PMC13362552; doi:10.3390/ma19132803)
Supplement: Supplementary file 1 [file materials-19-02803-s001.zip › materials-4370949-supplementary.pdf]

Supplementary data

Design-Oriented Comparison of Si–Me (Me = Mo, Ti, Zr, Ta, W) Infiltration Coatings on C/C Sonotrodes for Ultrasonic Atomization of CuSn8: Microstructure, Phase Constitution, Wettability, Nanoindentation, and Process Performance

Tomasz Choma [1,2], Mirosław Jakub Kruszewski [2,*], Aleksandra Chądzyńska [1], Bartosz Kalicki [1,2], Bartosz Morończyk [1], Jakub Ciftci [1,2], Łukasz Żrodowski [1,3], Joanna Zdunek [2] and Marcin Leonowicz [2]

[1] AMAZEMET Sp. z o. o. [Ltd.], 27 Jana Pawła II Ave., 00-867 Warsaw, Poland

[2] Faculty of Materials Science and Engineering, Warsaw University of Technology, 141 Wołoska St., 02-507 Warsaw, Poland

[3] Department of Materials Science & Engineering, Carnegie Mellon University, Pittsburgh, PA 15213, USA

* Correspondence: miroslaw.kruszewski@pw.edu.pl; Tel.: +48-22-234-8150; Fax: +48-22-234-8514

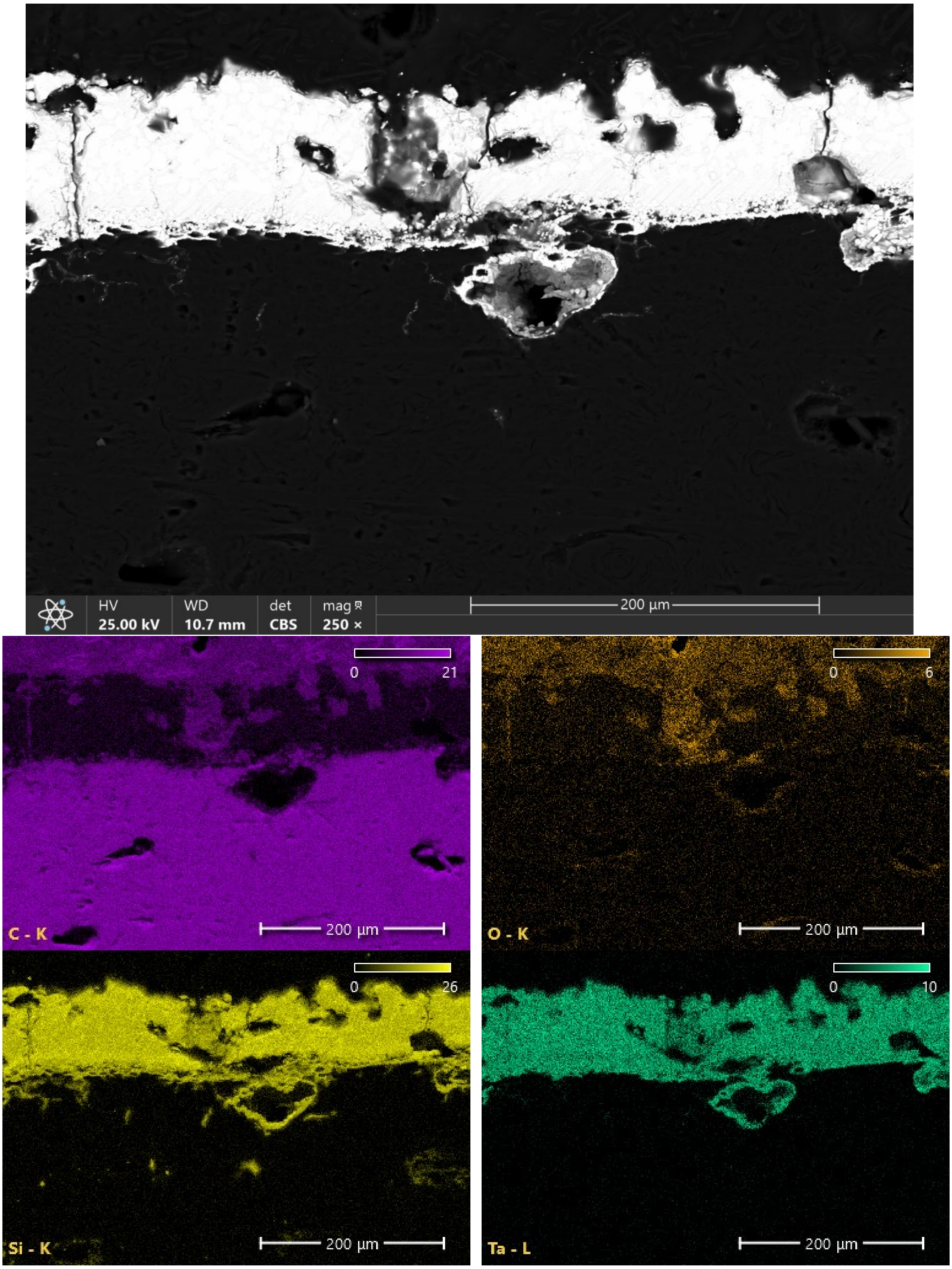

Figure S1 Elemental mapping Si:Ta 1:1 after infiltration

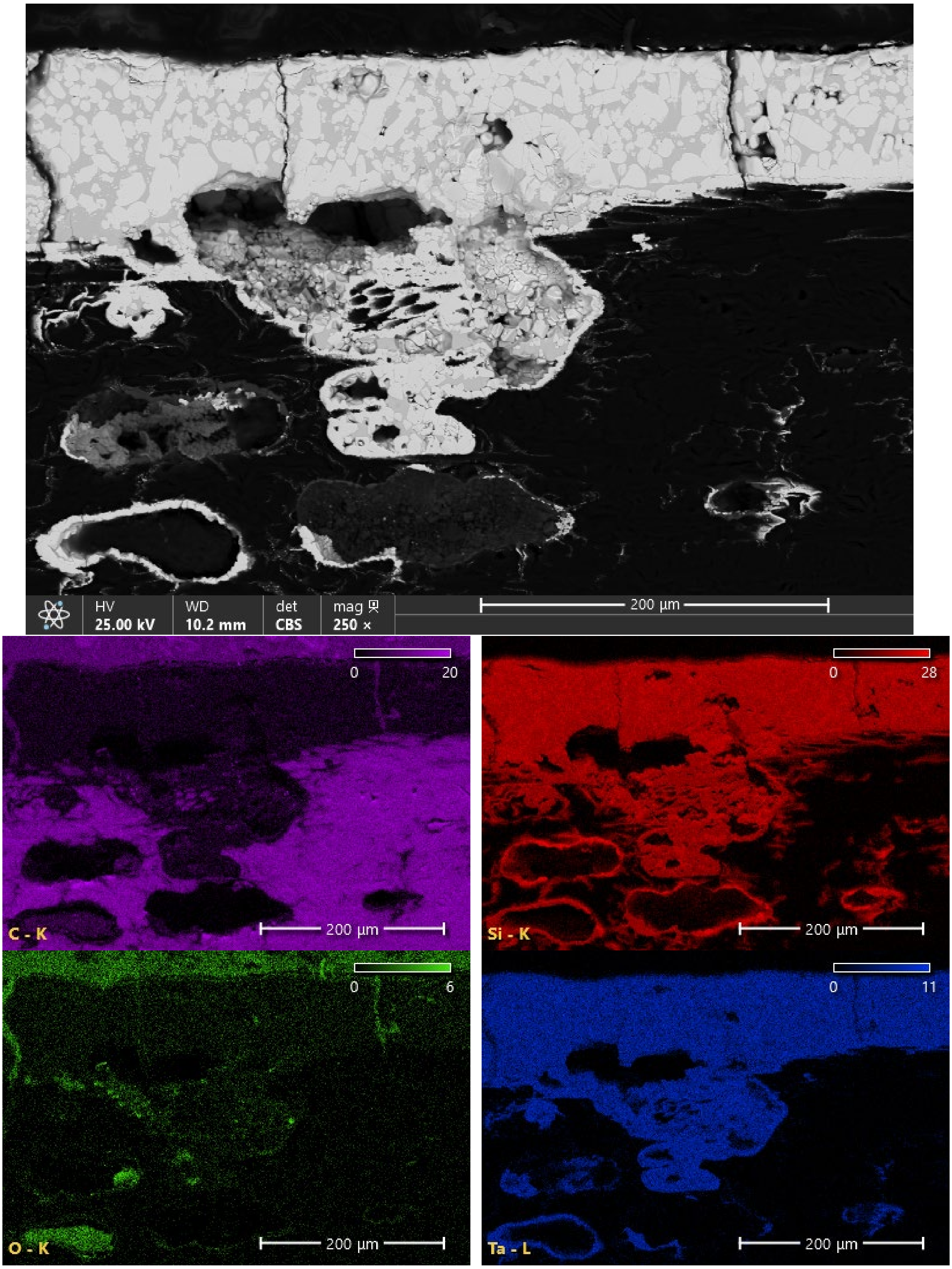

Figure S2 Elemental mapping Si:Ta 1:1 after atomization

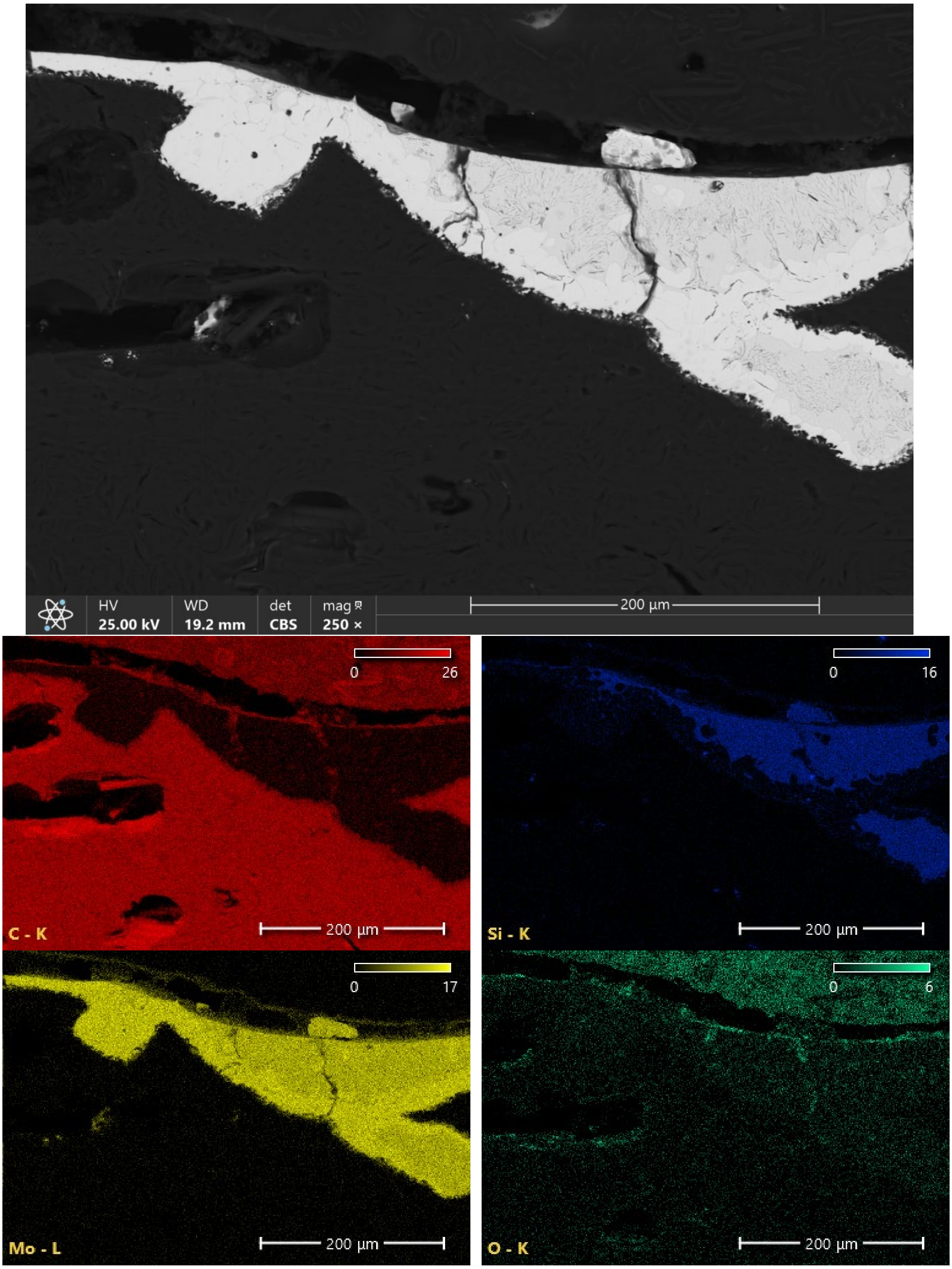

Figure S3 Elemental mapping Si:Mo 1:4 after infiltration

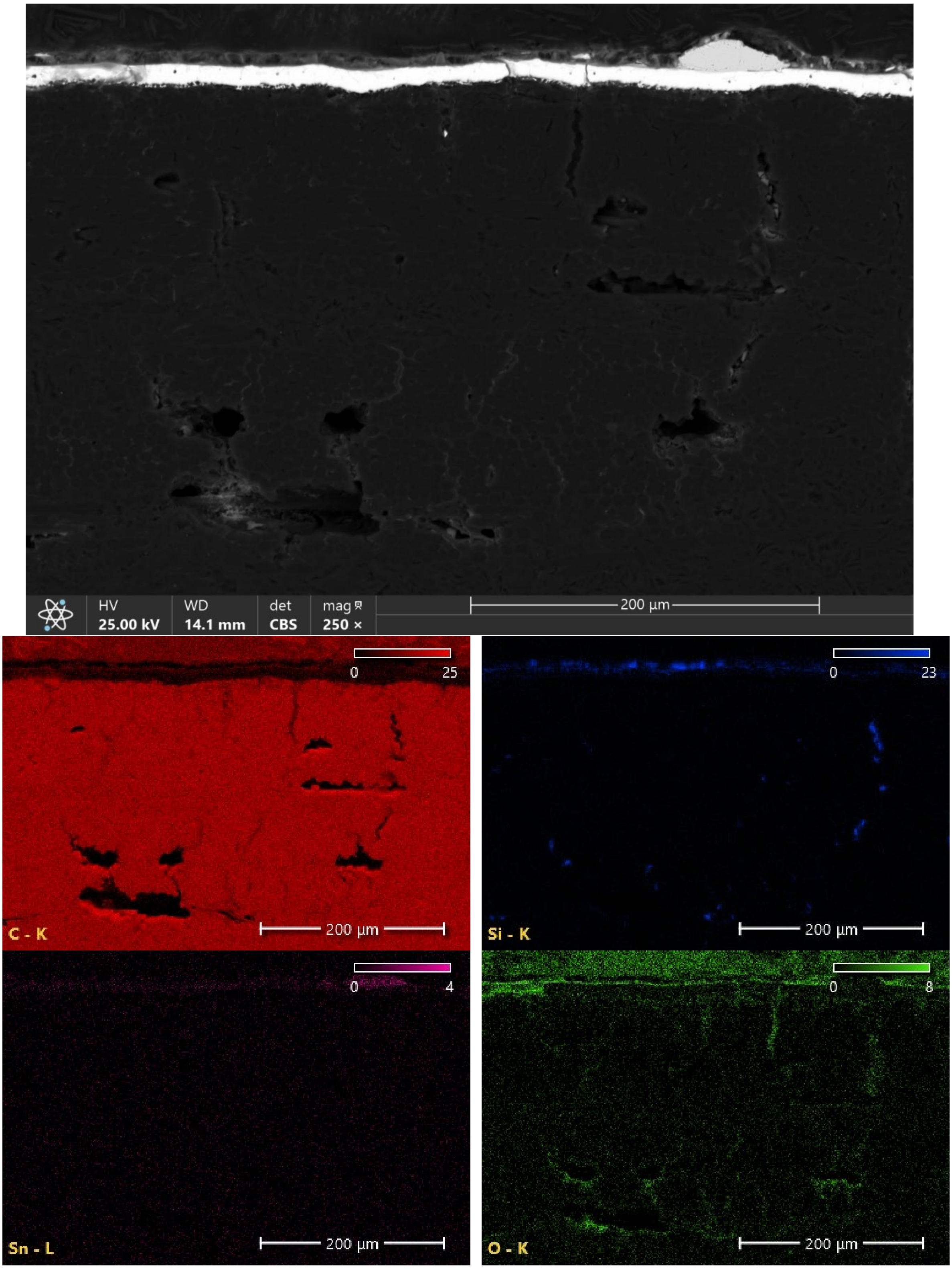
HV
25.00 kV
WD
14.1 mm
det
CBS
mag
250 ×
200 µm
C - K
0
25
200 µm
Si - K
0
23
200 µm
Sn - L
0
4
200 µm
O - K
0
8
200 µm

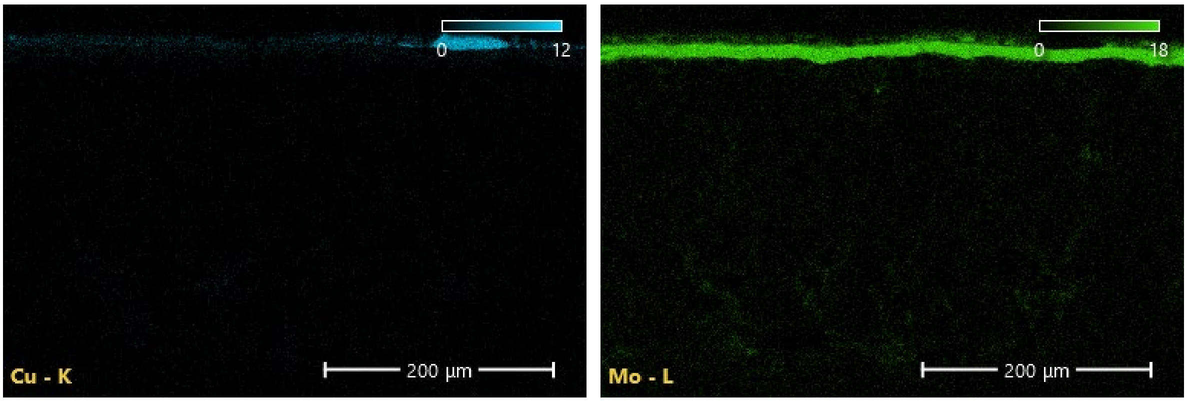

Figure S4 Elemental mapping Si:Mo 1:4 after atomization

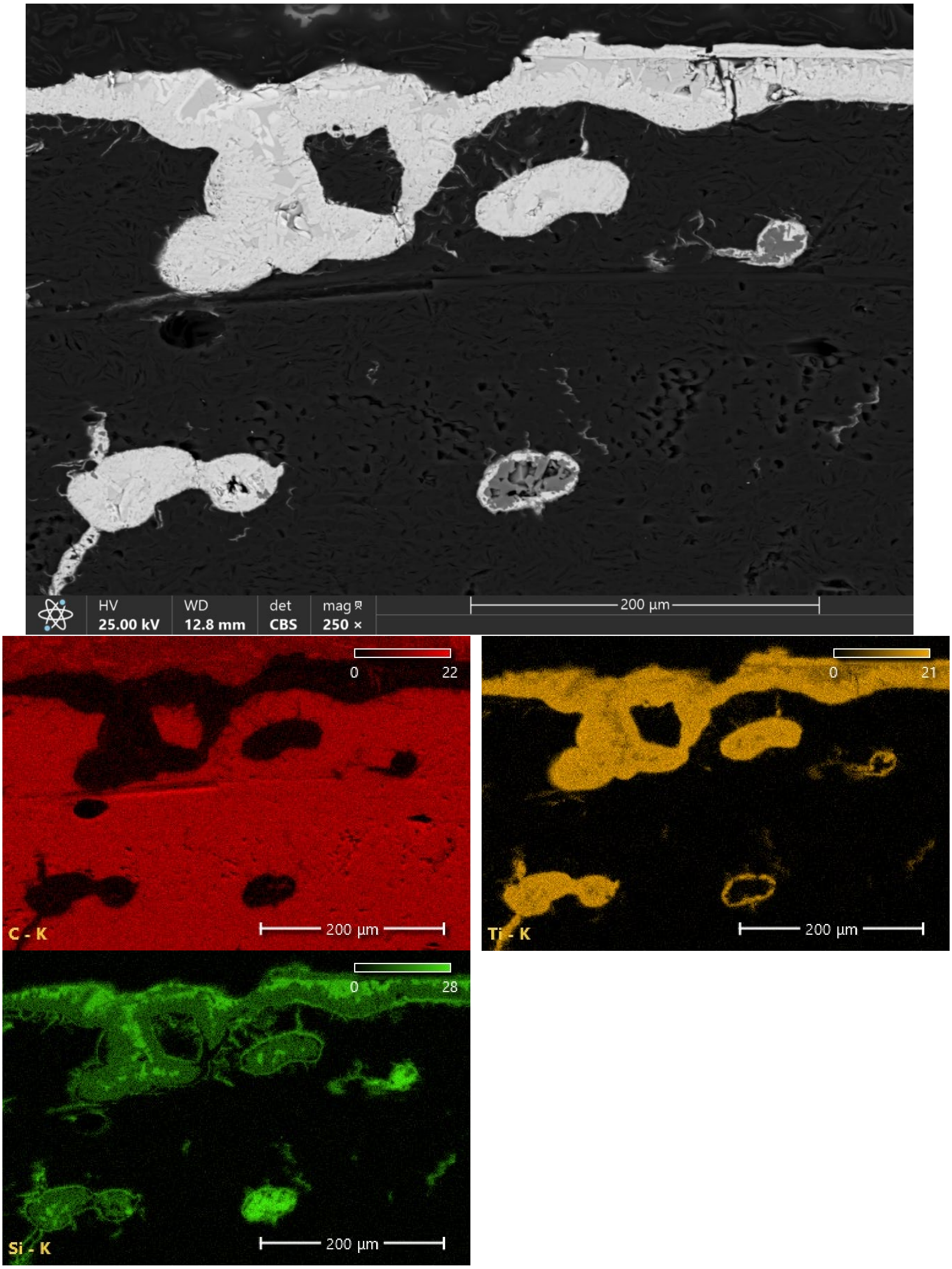

Figure S5 Elemental mapping Si:Ti 1:1 after atomization

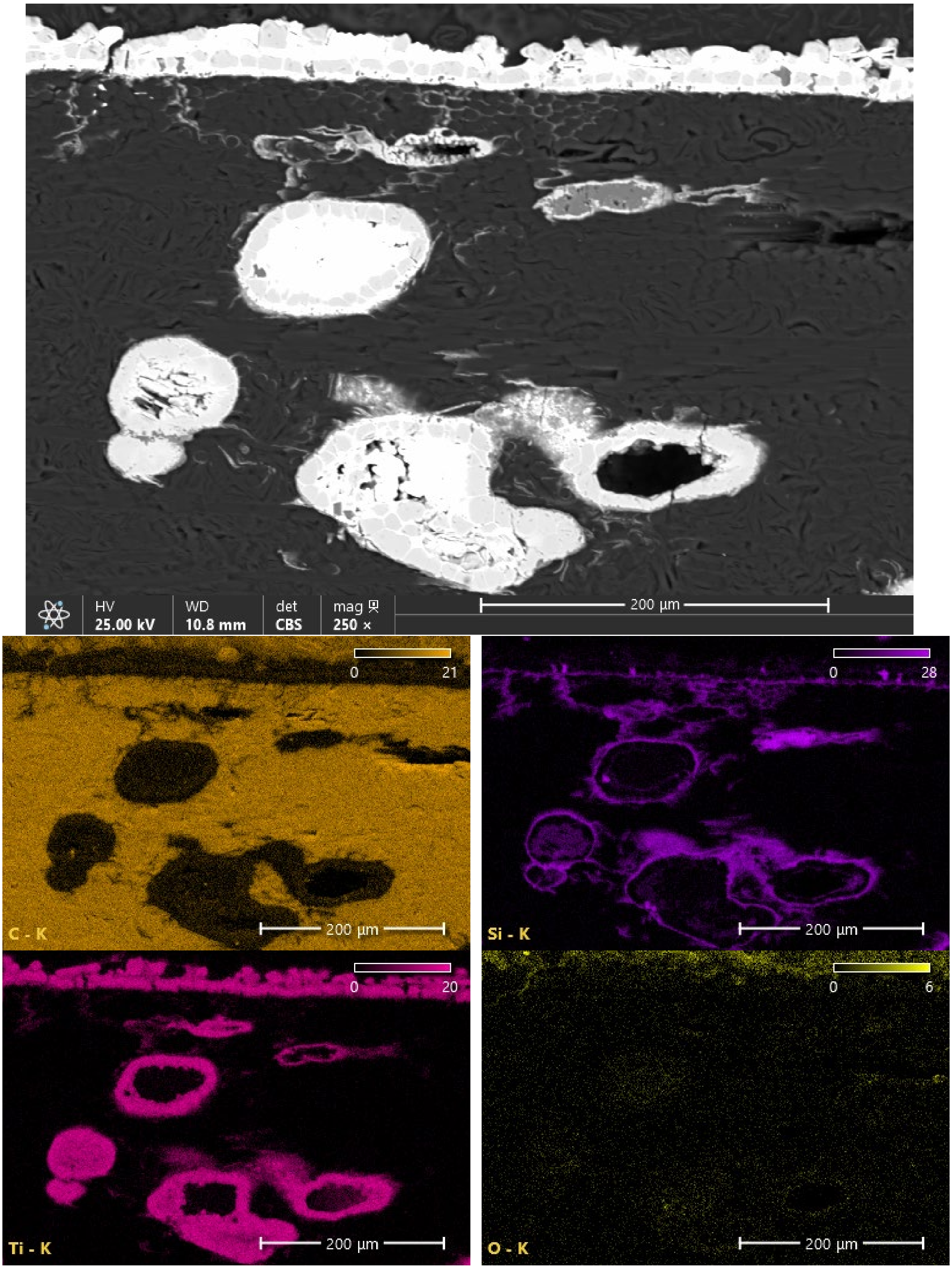
HV
25.00 kV
WD
10.8 mm
det
CBS
mag
250 ×
200 µm
0
21
C - K
0
28
Si - K
0
20
Ti - K
0
6
O - K

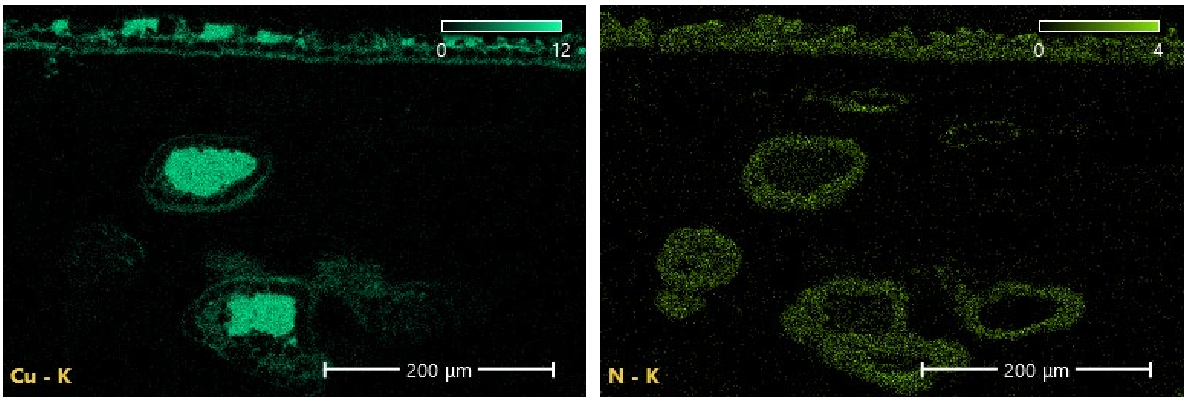

Figure S6 Elemental mapping Si:Ti 1:1 after infiltration

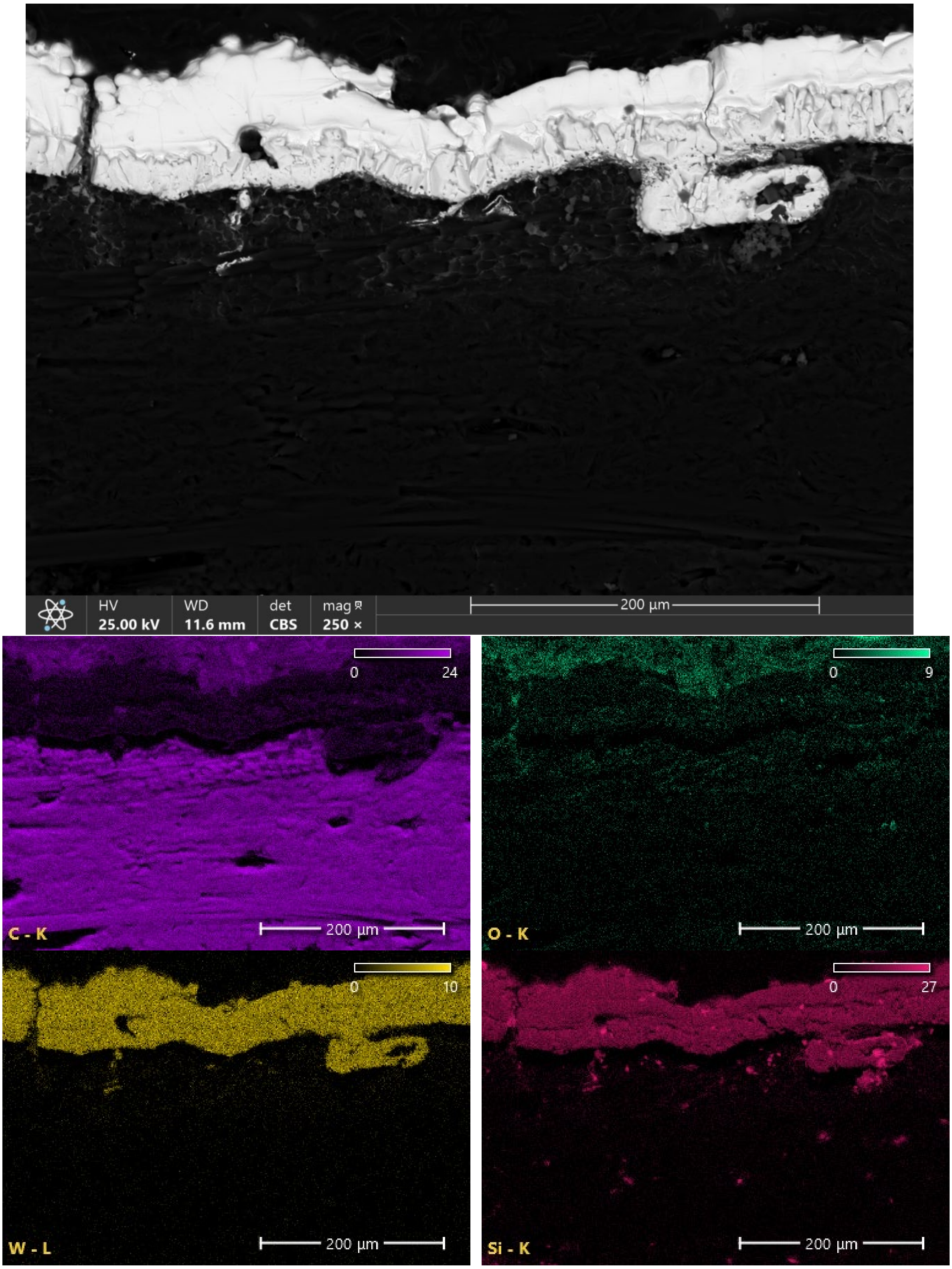

Figure S7 Elemental mapping Si:W 1:5 after infiltration

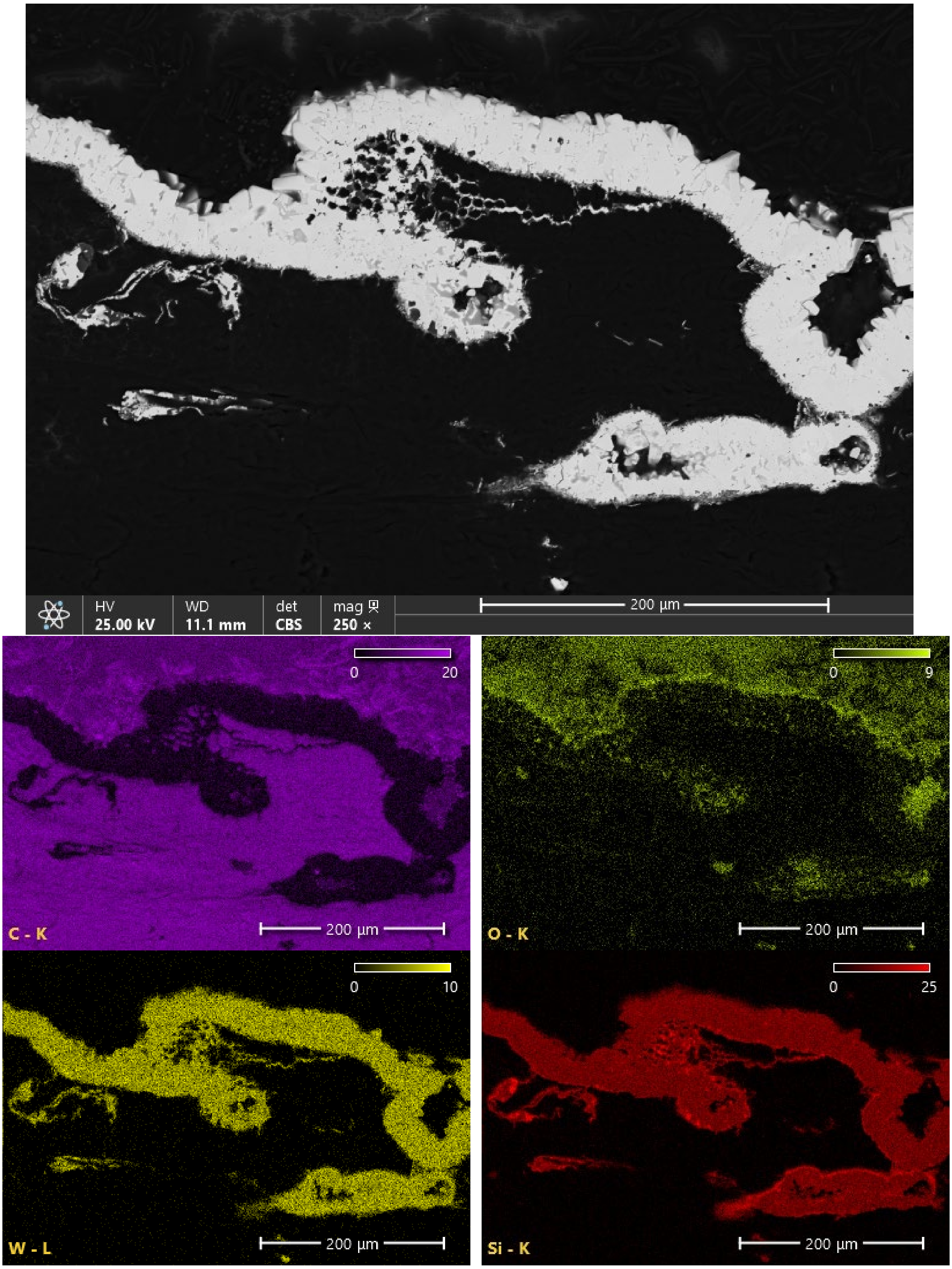

Figure S8 Elemental mapping Si:W 1:5 after atomization

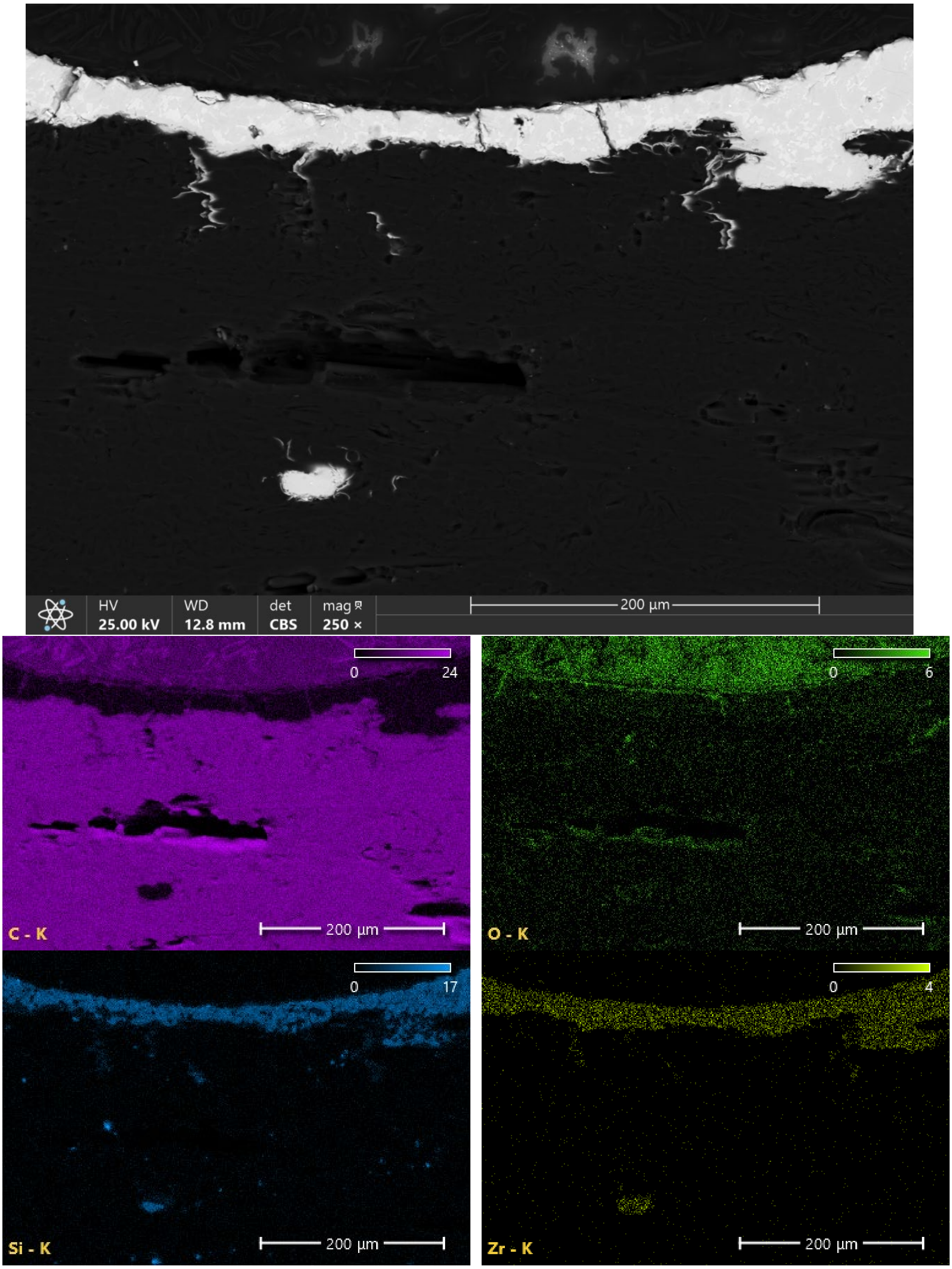

Figure S9 Elemental mapping Si:Zr 1:5 after infiltration

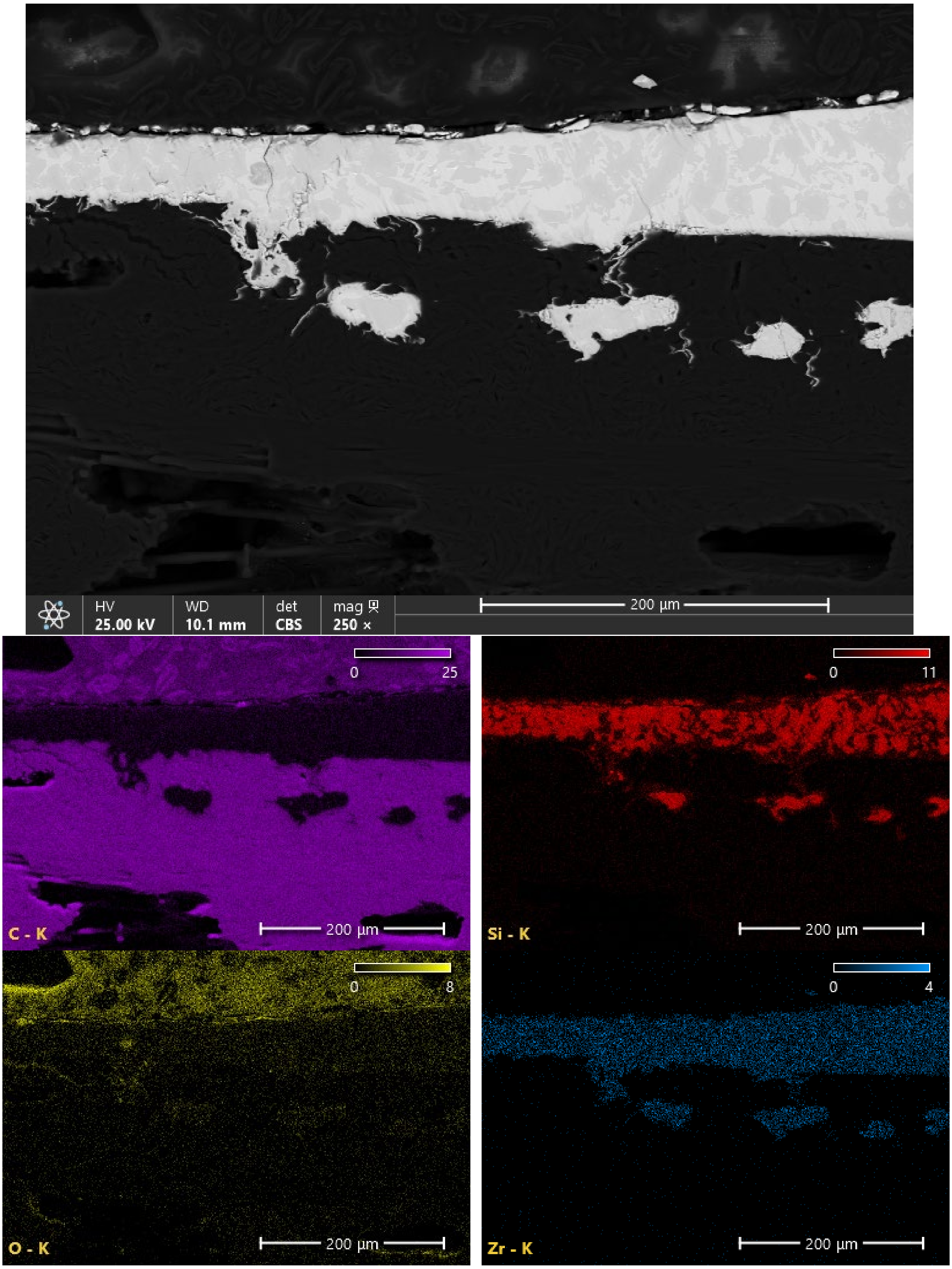

Figure S10 Elemental mapping Si:Zr 1:5 after atomization

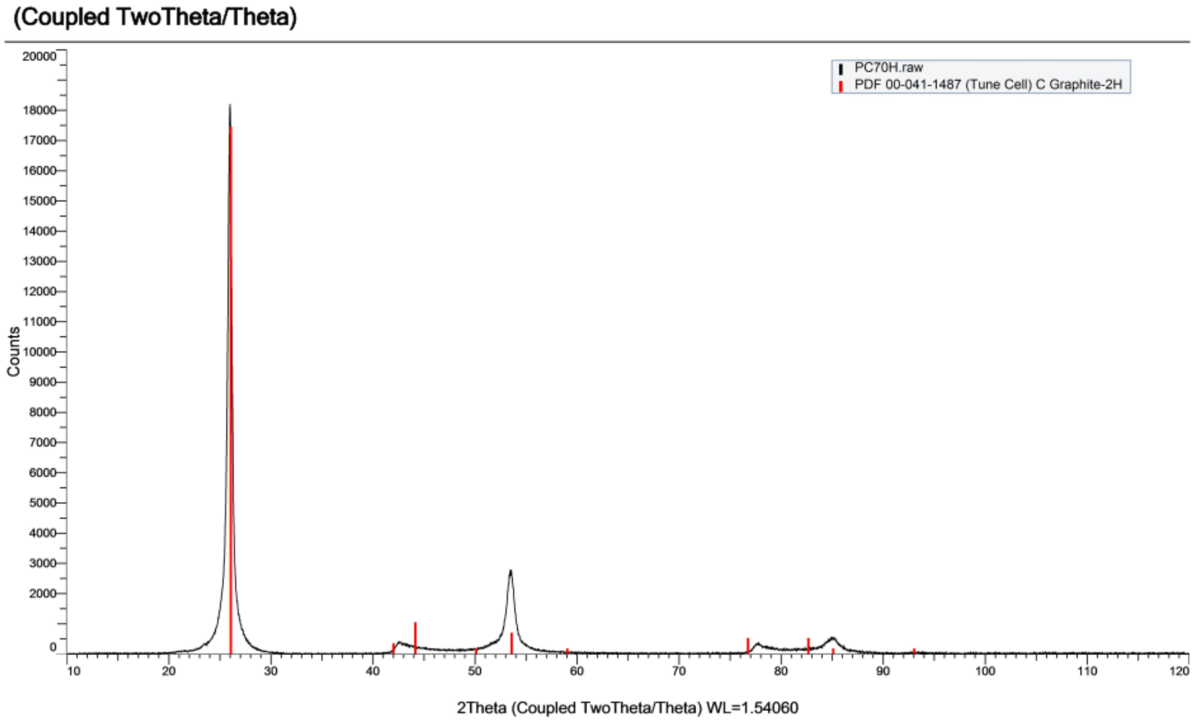

Figure S11 XRD patterns of C/C substrate before infiltration

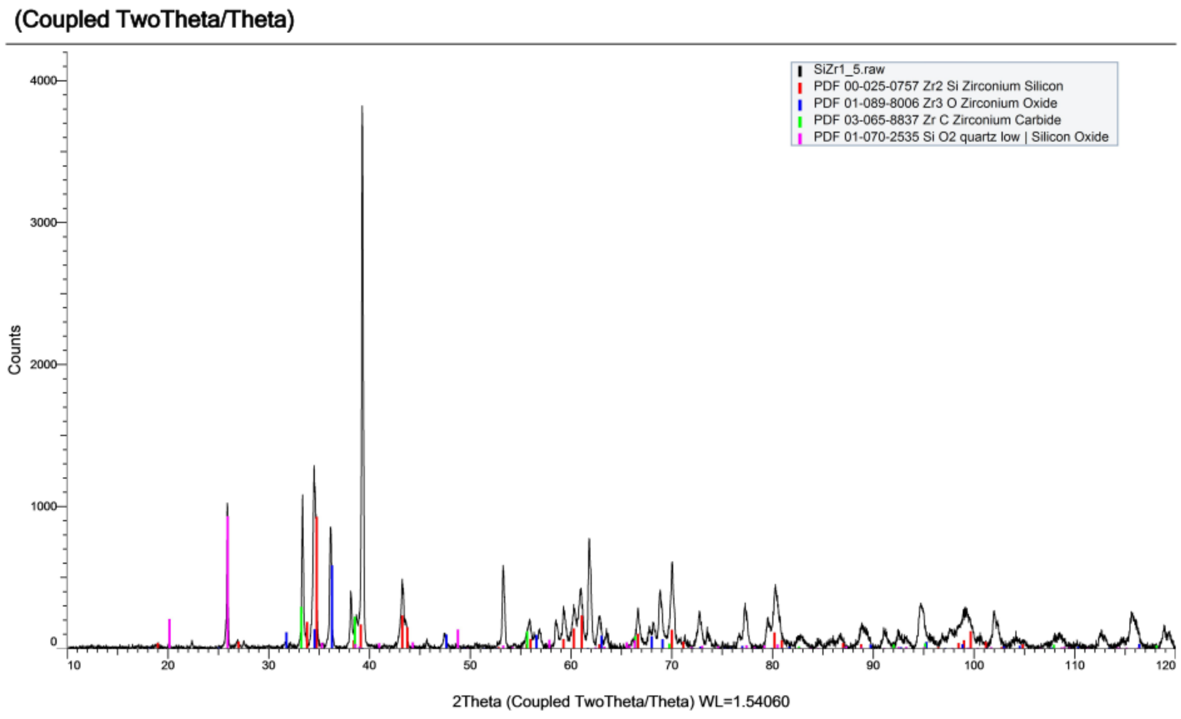

Figure S12 XRD patterns of Si:Zr 1:5 after infiltration

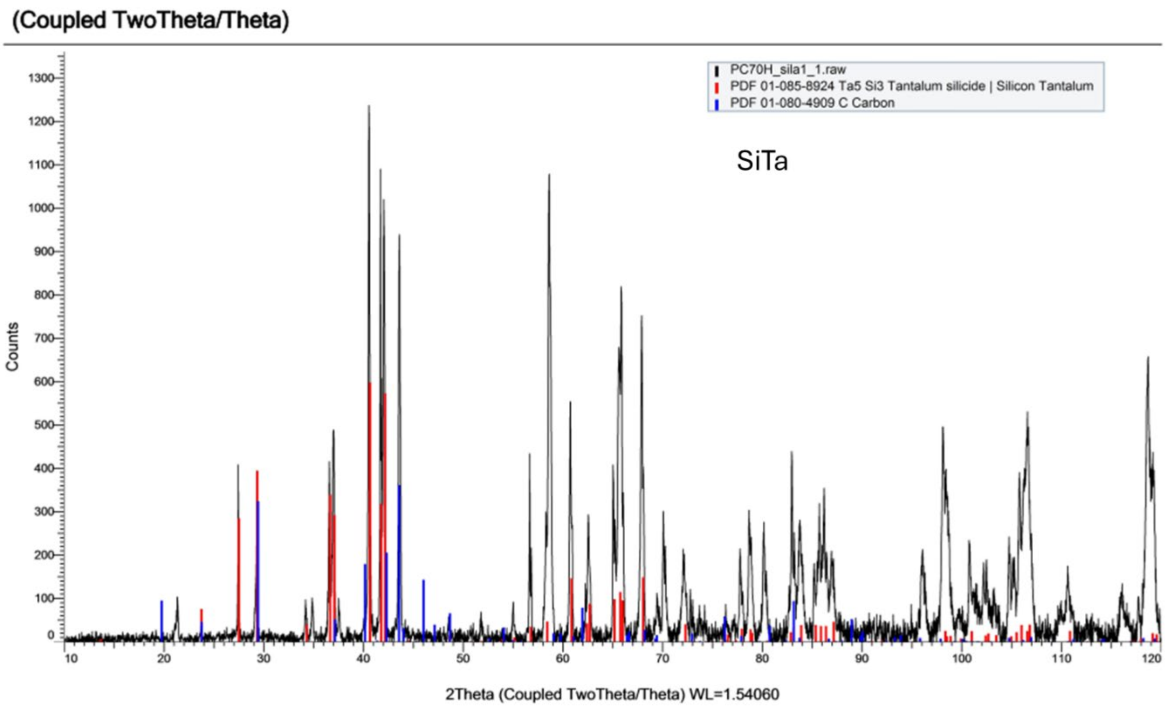

Figure S13 XRD patterns of Si:Ta 1:5 after infiltration

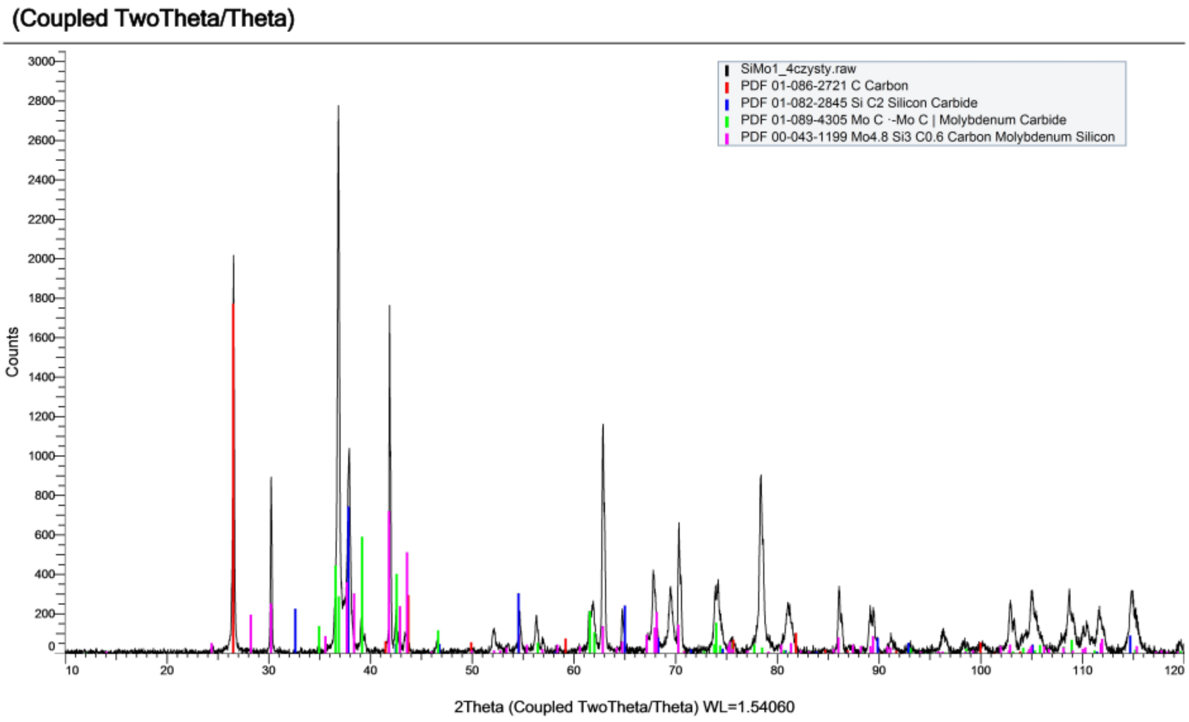

Figure S14 XRD patterns of Si:Mo 1:4 after infiltration

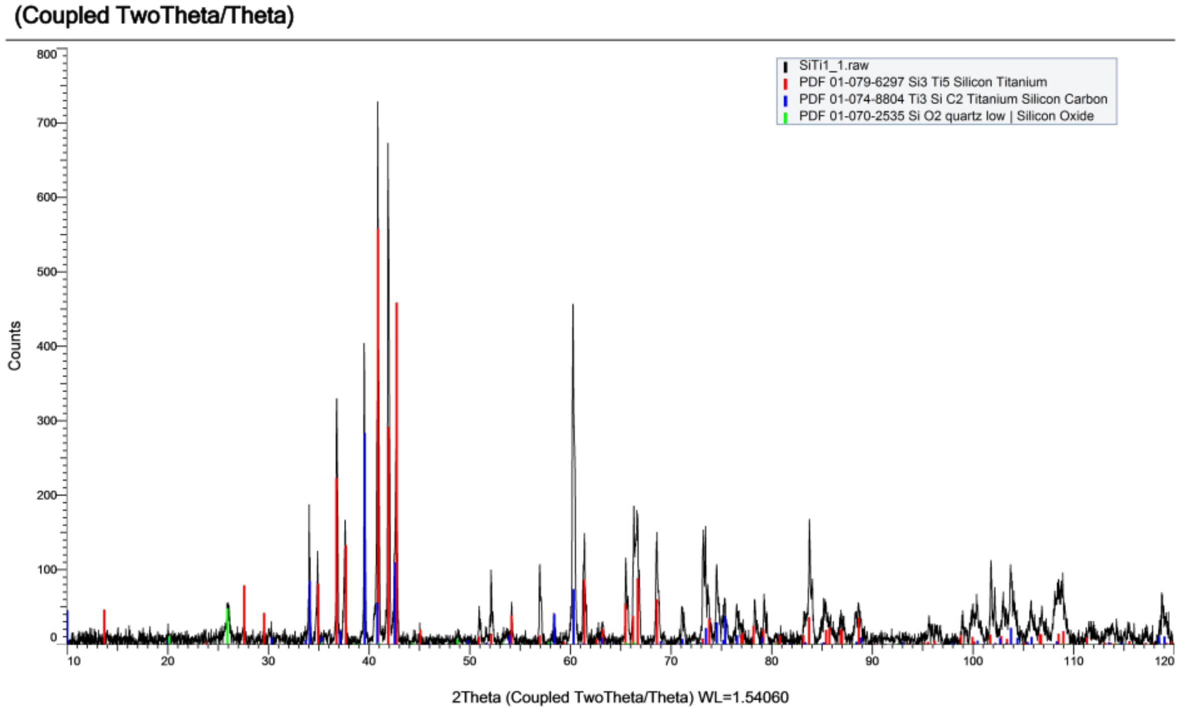

Figure S15 XRD patterns of Si:Ti 1:1 after infiltration

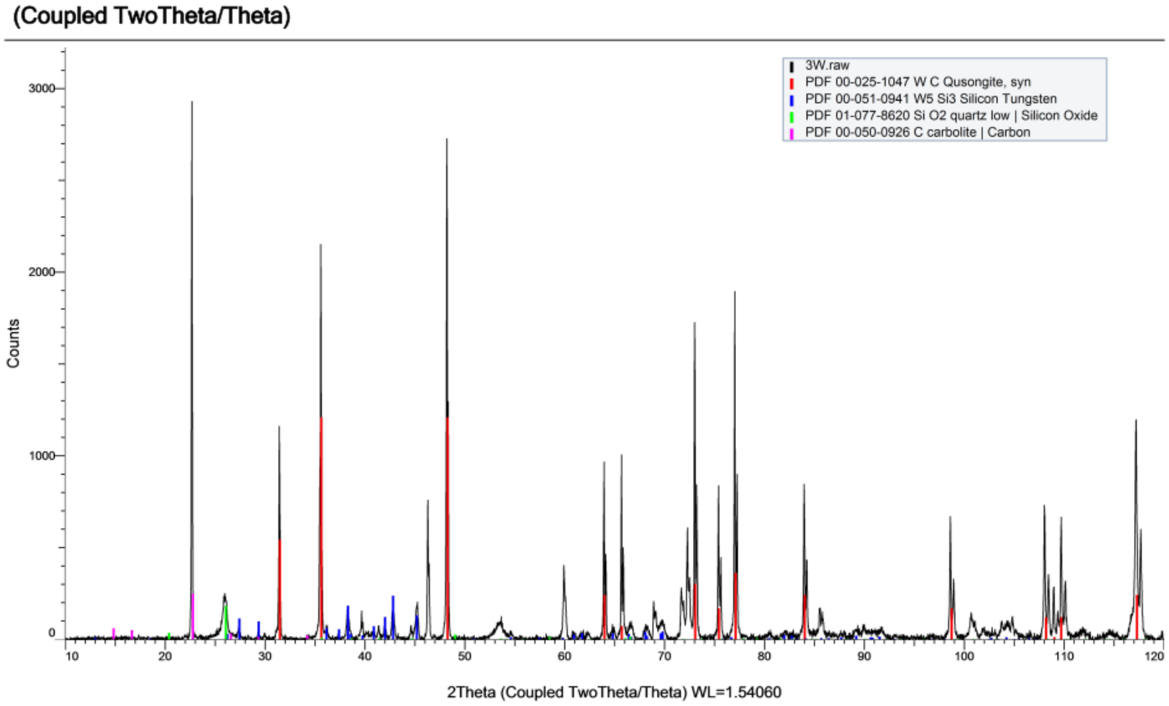

Figure S16 XRD patterns of Si:W 1:5 after infiltration
